# Facile Synthesis of Organically Synthesized Porous Carbon Using a Commercially Available Route with Exceptional Electrochemical Performance

**DOI:** 10.1021/acsami.4c09710

**Published:** 2024-08-26

**Authors:** Adam Rowling, Julien Doulcet, Robert Dawson, Nuria Tapia-Ruiz, Abbie Trewin

**Affiliations:** † Department of Chemistry, Lancaster University, Bailrigg, Lancaster LA1 4YB, U.K.; ‡ Department of Chemistry, University of Sheffield, Dainton Building, 13 Brook Hill, Sheffield S3 7HF, U.K.; § Department of Chemistry, Molecular Sciences Research Hub, White City Campus, Imperial College London, London W12 0BZ, U.K.

**Keywords:** conjugated microporous polymers, porous materials, amorphous materials, acetylene frameworks, anode materials, lithium ion batteries

## Abstract

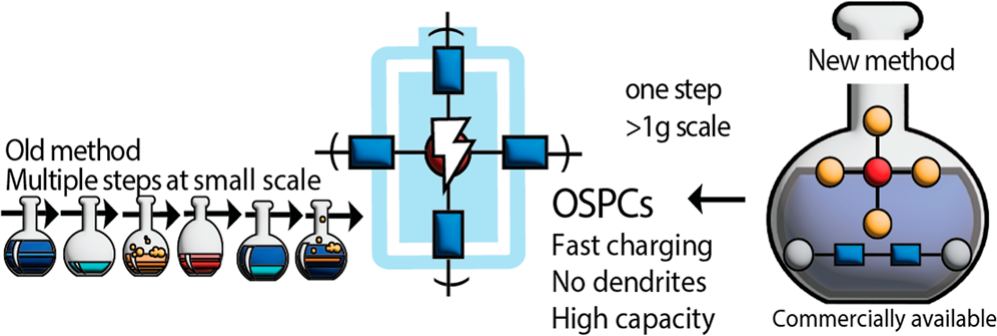

Organically synthesized porous carbon (OSPC) is a subclass of conjugated microporous polymer materials that have shown potential applications as anodes in ion batteries. However, a challenging, low-yielding, multistep synthetic route (the A method) has hindered further exploration of this exciting family. Here, OSPC-1 has been synthesized via an alternative, efficient one-pot method from commercially available reagents (the B method), hereafter referred to as OSPC-1b in contrast to OSPC-1a, where it is synthesized via the A method. Characterization revealed the same polymer structure and the highest surface area to date of an OSPC (or OSPC analogue) family member for OSPC-1b with 909 m^2^ g^–1^. OSPC-1b was tested as an anode for Li-ion batteries, demonstrating the same high capacity, fast charging, resistance to degradation, and inhibition of the formation of dangerous lithium dendrites as OSPC-1a. Furthermore, the electrochemical properties of OSPC-0 were evaluated for the first time, agreeing with previously predicted values, giving scope for the design and targeting of specific properties.

## Introduction

Microporous organic polymers (MOPs) are a diverse class of materials that exhibit excellent stability and versatile functionality. MOPs encompass several subclasses including hyper-cross-linked polymers (HCPs),^1^ conjugated microporous polymers (CMPs),^2,3^ polymers of intrinsic microporosity,^4^ and covalent triazine-based frameworks.^5^ The wide range of synthetic strategies for generating MOPs allows for the development of robust and tailored properties for specific applications.

CMPs are an important subclass of MOPs with applications across many areas including gas uptake,^6^ solid-state electrolytes for fuel cell technologies,^7^ and as a nanoporous supercapacitor.^8,9^ The amorphous three-dimensional framework of CMPs contains microporosity originating from inefficient packing of the polymer chains. This combination of high porosity and electronic activity makes CMPs promising materials for energy storage and conversion technologies.

**Figure 1:**
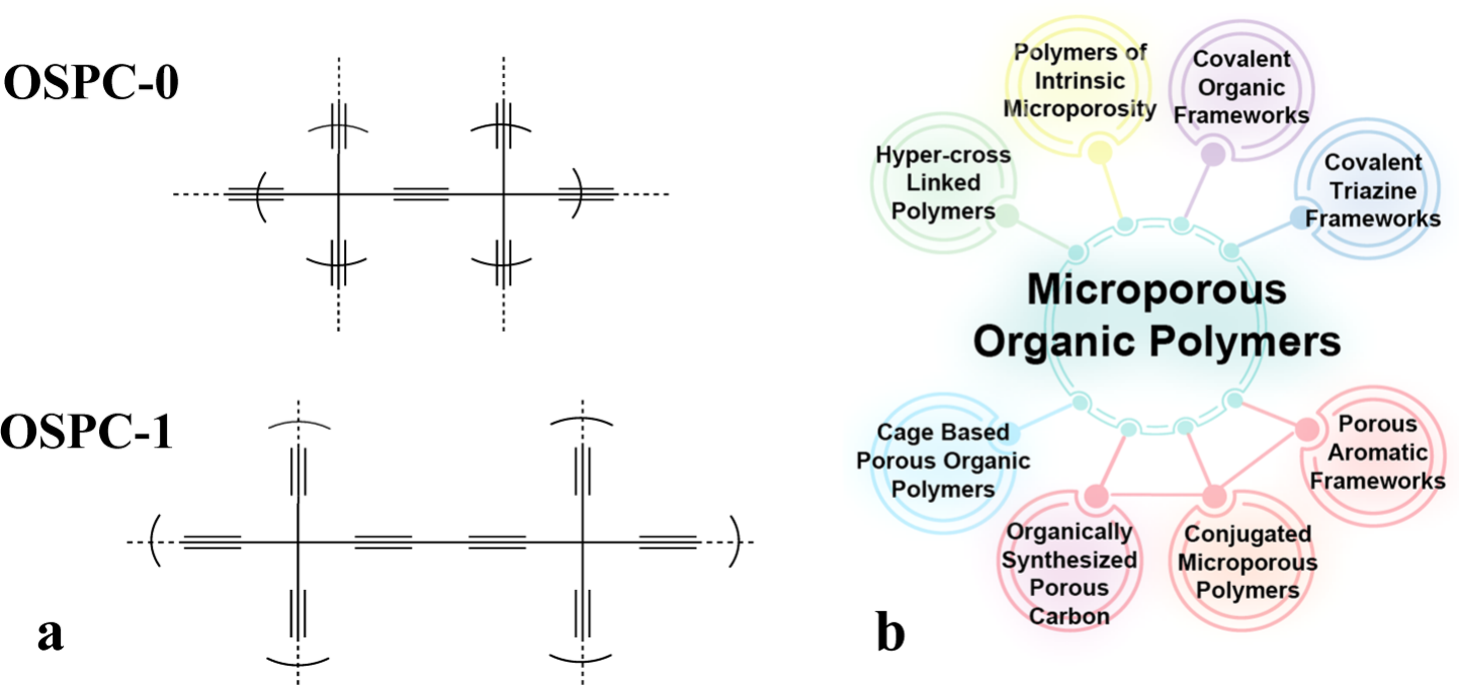
(a) Chemical repeat structure of OSPC polymer materials, OSPC-0 and OSPC-1. (b) How OSPCs fit into the family of MOP materials.

A new member of the conjugated microporous polymer family is organically synthesized porous carbon one (OSPC-1)^10^ constructed entirely from sp^3^ carbon nodes linked via sp carbon atoms in a three-dimensional network; the chemical structure is shown in Figure 1a. While the framework is built entirely with carbon atoms, like diamond or graphite, its synthesis and properties are closer to a porous polymer, and so, we place it firmly within this family, as shown in Figure 1b.

Organically synthesized porous carbon (OSPC) generated significant interest due to its performance as an anode in lithium-ion batteries (LIBs). Though it demonstrated a considerably greater capacity for energy storage than graphite at both low and high current densities, it drew the most attention for its high resistance to degradation upon cycling. Even after cycling at currents as high as 3000 mA g^–1^ for 500 cycles, no reduction in capacity and, crucially, no formation of lithium dendrites was observed in the OSPC-1-based cells, whereas both were seen in the conventional graphite half-cells. This was a significant finding because dendrite formation in commercial LIBs is a major safety concern.^11,12^ Furthermore, many new anode materials designed to outperform graphite suffer from poor cycling stability.^13^ An expansion of the OSPC family of materials has been explored through computational methods,^14,15^ with several family members showing potential for exceptional electrochemical properties. These include an increase in the predicted lithium-ion storage capacity but, importantly, also include family members with predicted high lithium diffusion properties, a key challenge for producing fast-charging batteries. Silicon- and germanium-based analogues of OSPC-1 have recently been published.^16,17^ The tetraethynylsilane and germane monomers are substantially more stable than the substituted methane monomer used to make OSPC-1, although they still require multiple synthetic steps under an inert atmosphere.^16,17^

**Figure 2:**
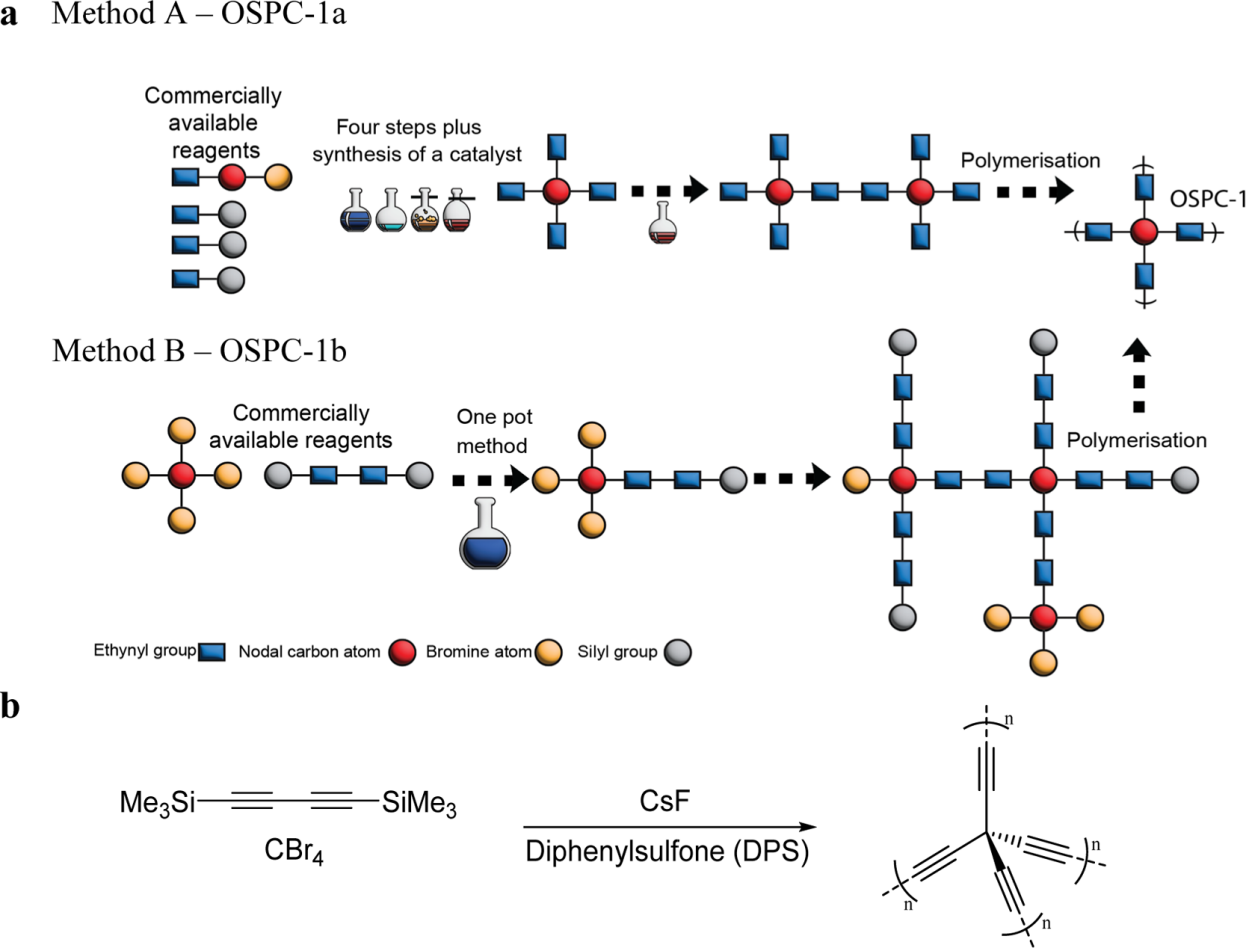
(a) Schematic of the multistep Method A to make OSPC-1a and the one-pot Method B to produce OSPC-1b and (b) reaction scheme for Method B for OSPC-1.

However, there have been no reports of the synthesis of OSPC-1 since the initial publication. This is likely due to the complexity of the low-yielding six-step synthetic procedure reported to access OSPC-1, hereafter referred to as the multistep method (Figure 2, Method A, OSPC-1a). In contrast, synthesis of the single acetylene strut 3-D porous carbon (analogous to a predicted OSPC family member named OSPC-0) was recently reported via a simple one-pot method from commercially available materials, hereafter referred to as the one-pot method.^18^

In this work, we show that the method reported to synthesize OSPC-0 can be extended to the synthesis of OSPC-1 (OSPC-1b, Figure 2, Method B) and that OSPC-1’s electrochemical properties are preserved despite the greatly simplified synthesis. We also expand the electrochemical testing to OSPC-0, which also shows electrochemical properties that are in line with the properties predicted through computational methods.^15^ This, therefore, opens the OSPC family of materials for further exploration of their exciting potential as electrode materials and further provides a route for their commercial application.

## Results and Discussion

### Structural Properties of OSPC-0b and OSPC-1b

OSPC-0 and OSPC-1 have been synthesized here according to Method B, the one-pot synthesis, outlined in the Supporting Information, and purified using standard methods.^18^ They are hereafter named OSPC-0b and OSPC-1b, respectively. Although both materials share the same short-range node-strut structure, given the different polymerization methods used, the respective networks grow through different pathways and have different terminating end groups. OSPC-1a is terminated with hydrogen end groups, whereas OSPC-1b is terminated with a mixture of bromine atoms and silyl groups, which may influence the resulting electrochemical properties (as shown in Figure 2). Further, the properties of chemically identical microporous polymers can vary significantly when made in different solvents and even more when made through different cross-coupling reactions.^19,20^ An OSPC-1 network made by attaching nodes to struts one by one, as happens for the one-pot Method B, could therefore have vastly different properties from one made by joining presaturated nodes together, as happens for the multistep Method A.

To assess the influence of the solvent on Method B, we first used diphenyl sulfone (DPS), as originally reported, and a range of alternate solvents were considered, as shown in Table S1. As DPS is solid below 128 °C, difficulties were encountered during extraction and purification of the product. However, all alternative solvents tried had lower boiling points, which could be responsible for the lower yields observed when compared to DPS under otherwise identical conditions. It was concluded that DPS was optimal and so was used in the synthesis of all OSPC samples described here.

Calculating the yield of OSPC materials is challenging. The theoretical product comprises carbon alone, so the presence of unreacted end groups in the observed structure would lead to the observed yield being greater than the theoretical. Thus, a direct comparison of chemical yields between methods A and B is difficult. The overall yield of the longest linear sequence of steps for method A is 17%, which excludes the synthesis of chlorotriethylsilylacetylene, a major component of the monomer synthesis.

Using method B, gram quantities of OSPC-1b were obtained in a single step from tetrabromomethane and 1,4-bis(trimethylsilyl)butadiyne in high yields (84% based on the abundance of noncarbon elements observed at the surface of OSPC-1b by XPS, see Section S10). This represents a significant improvement over method A, and considering the prevalence of end groups is likely to be greater at the surface than in the bulk structure we can consider this to be a lower bound. As described in Section S4.5, OSPC-1b is at least 20 times less expensive to synthesize than OSPC-1a in raw materials alone, and the simplicity of the method (no inert atmosphere required, single reaction vessel) offers significant potential for scaling up to commercial production.

**Figure 3:**
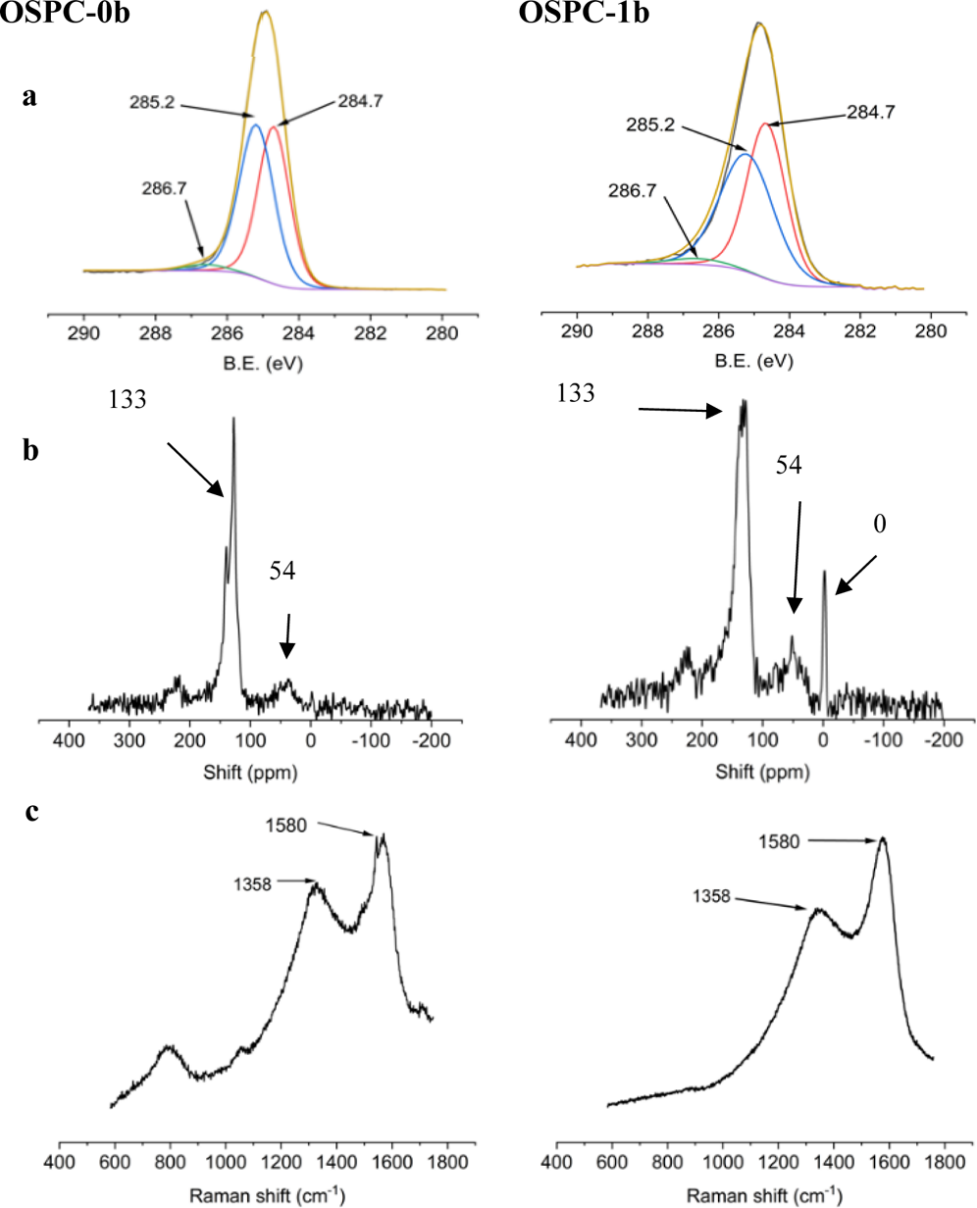
Characterization data of OSPC-0b (left) and OSPC-1b (right). (a) Fitted C 1s XPS spectra. The peak at 284.7 eV is assigned to sp^3^ carbon, the peak at 285.2 eV is assigned to sp carbon, and the peak at 286.7 eV is assigned to carbon bonded to bromine end groups. (b) Solid-state ^13^C NMR spectra with peaks labeled; nonlabeled peaks are spinning sidebands, as confirmed by analysis at a higher spinning speed shown in Figure S3 and (c) Raman spectra.

The amorphous and insoluble nature of these materials makes their structural characterization challenging, and so, a holistic approach of relevant characterization and computational techniques was used from which the structure can be inferred. For porous amorphous materials, solid-state NMR (ssNMR) and or IR/Raman were used to confirm the short-range chemical structure, and gas sorption analysis was used to assess the porosity.^21−25^ XPS analysis was used for OSPC-1a to confirm the presence of only sp- and sp^3^-hybridized carbon atoms within the structure. XPS was undertaken here on the resulting OSPC-0b and OSPC-1b samples, with a summary shown in Figure 3a and the full details in Section 10 of the Supporting Information. The C 1s peak at 285 eV was common to all spectra and was deconvoluted into three subpeaks. In accordance with previously reported data, the peak at ≈284.7 eV was assigned to a carbon node with sp^3^ hybridization, the peak at ≈285.2 eV was assigned to a carbon strut with sp hybridization, and the peak at ≈286.7 eV was assigned to carbon bonded to the Br end groups. As previously observed with OSPC-1a, the differences between the observed and expected ratios of C sp^3^ and C sp can be explained by the elevated abundance of end groups and greater exposure at the material’s surface. Survey XPS spectra were collected for OSPC-0b and OSPC-1b, which reported elemental abundance percentages in line with expectations (Tables S5 and S6). ssNMR spectra of OSPC-0b and OSPC-1b are shown in Figure 3b and matched well with previously reported spectra.^10,18^ For OSPC-0b, we observed a split peak at ca. 133 ppm, which we attribute to the acetylene C sp struts. The splitting is due to the acetylene strut within the bulk of the polymer network being more shielded, while the acetylene C sp struts at or near the polymer network’s surface are less shielded. The peak at 54 ppm was assigned to the C sp^3^ nodes. Similarly, for OSPC-1b, we assign the peak at 133 ppm to acetylene C sp struts, and the peak at 54 ppm is assigned to the C sp^3^ nodes. The peak at 0 ppm is assigned to residual trimethylsilyl protecting groups from the monomer, as suggested by the XPS wide data (Figure S10).

**Figure 4:**
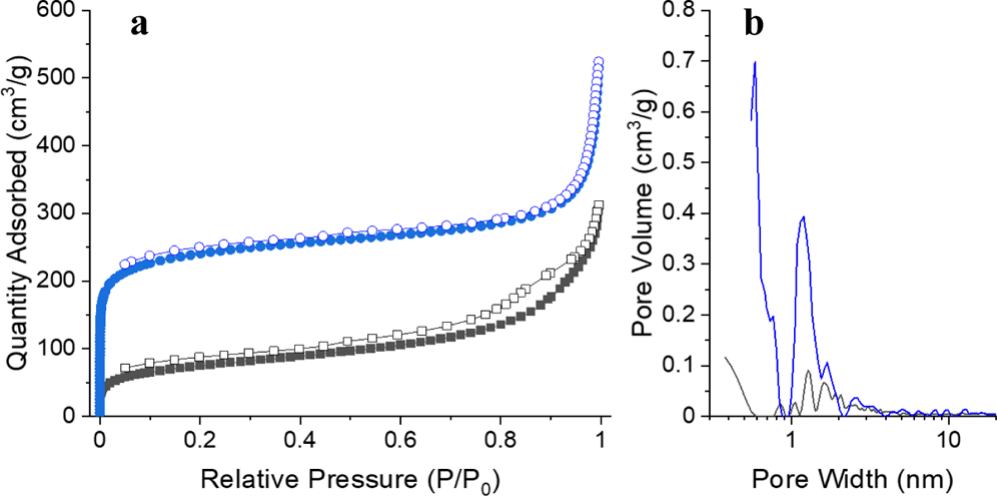
(a) Nitrogen uptake isotherms and (b) pore size distribution plots for OSPC-1b (blue) and OSPC-0 (black). Surface areas of 909 and 261 m^2^ g^–1^ were obtained from the analysis of the OSPC-1b and OSPC-0 uptake isotherms shown here, respectively.

Raman spectroscopy data of OSPC-0b and OSPC-1b in the 600–1800 cm^–1^ range are shown in Figure 3c, and wide-range data are found in Figure S4. Both materials showed two distinct peaks at 1358 and 1580 cm^–1^, assigned to C sp stretching, similar to those observed in OSPC-1a,^10^ further corroborating the XPS data. Although peaks appear at similar locations to the D and G peaks reported in graphite and graphitic materials, it is not appropriate to assign them as such as they originate from different Raman interactions.^26,27^ OSPC-0 has an additional peak at 800 cm^–1^ which, following computational analysis detailed in Section S6, we attribute to sp^3^–sp stretching at the node centers. Scanning electron microscopy (SEM) images of OSPC-0b and OSPC-1b showed similar microstructural properties to those materials previously published and are shown in Figure S11.^10^ The nitrogen uptake isotherms were collected for two samples of both OSPC-0b and OSPC-1b and are type II/IV in appearance, as shown in Figures 4 and S8. Analysis of the nitrogen uptake isotherm for OSPC-1b using the BET equation gave surface areas of 726 and 909 m^2^ g^–1^, similar to that reported for OSPC-1a (766 m^2^ g^–1^) and for the silicon node OSPC analogue (779 m^2^ g^–1^).^10,16^ The germanium node OSPC analogue was synthesized as a thin film on copper, so BET surface area data were not reported.^17^ For OSPC-0b, analysis of the nitrogen uptake isotherm using the BET equation reported surface areas of 261 and 473 m^2^ g^–1^, in agreement with the value predicted through computational simulation.^14,15^ CHNS analysis and EDX were also performed, with data reported in Sections S9 and S11 in Supporting Information.

### Electrochemical Properties of OSPC-0b and OSPC-1b

**Figure 5:**
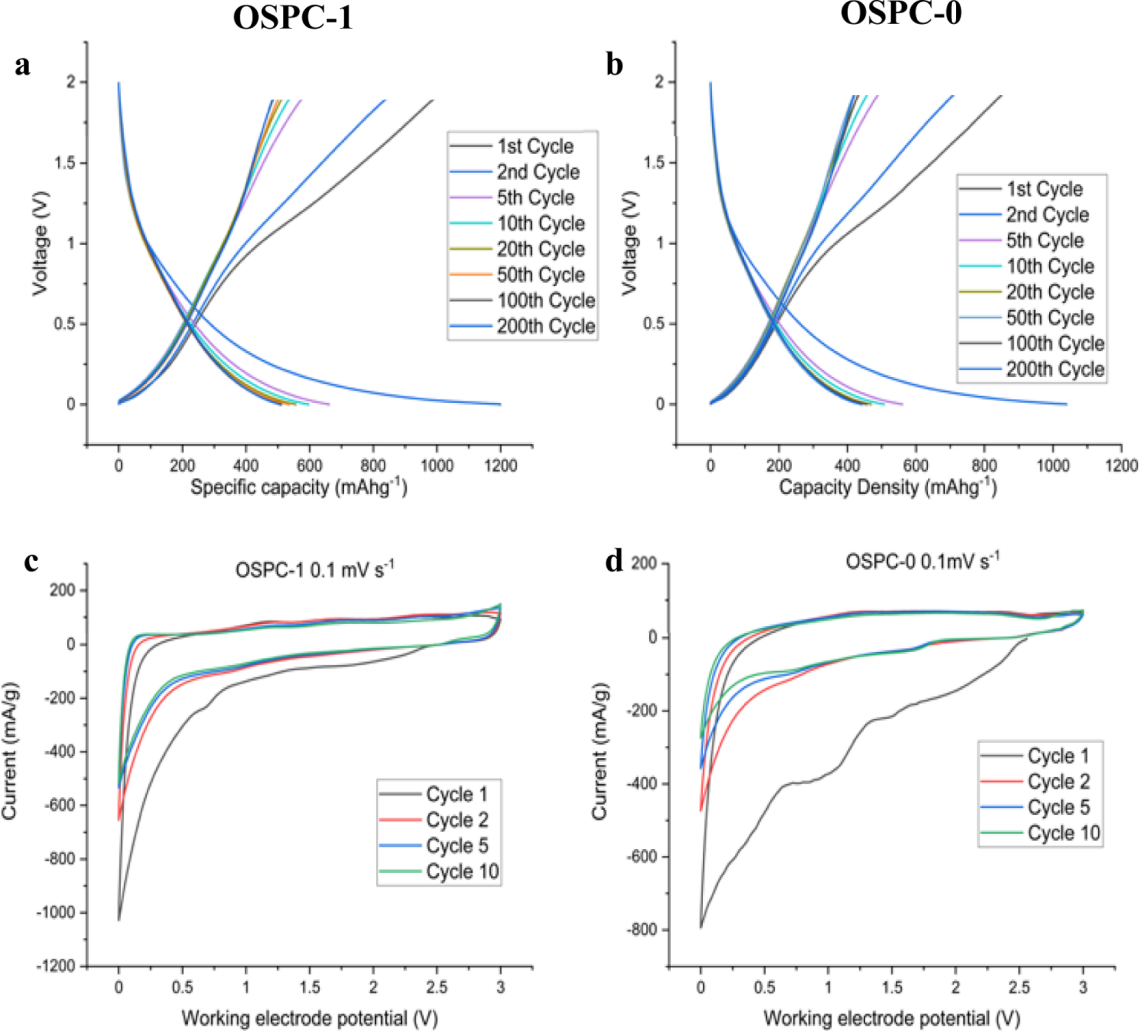
GCD cycling of (a) OSPC-1b and (b) OSPC-0b in the voltage window of 0–2 V at a current density of 200 mA g^–1^. The first discharge is omitted for clarity; unedited plots are shown in Figure S13. CV data for OSPC-1b (c) and OSPC-0b (d) between 0 and 3 V, scanning at 1 mV s^–1^.

Coin cells with OSPC-1 and OSPC-0 electrodes were prepared according to section S1.7, with each test run on different cells.^34^ Galvanostatic charge/discharge (GCD) cycling in the 0–2 V voltage window at 200 mA g^–1^ of OSPC-1b and OSPC-0b anodes is shown in Figure 5a,b, respectively. Large initial discharge capacities of 4000 and 3500 mA h g^–1^, attributed to solid electrolyte interphase (SEI) formation, were observed for both materials.^10^ The observed irreversible initial capacities (ICE) corroborate the larger currents with respect to the subsequent cycles observed in the cyclic voltammetry (CV) data, as shown in Figure 5c (OSPC-1b) and Figure 5d (OSPC-0b). After the first charge, the voltage profiles of OSPC-1b and OSPC-0b showed reversible slopes upon cycling. We should note, however, the presence of two cathodic peaks at 1 and 1.5 V in the first sweep for OSPC-0b, which shifted to 0.7 and 1.7 V, respectively, and are not reversible in subsequent cycles.

The load curves and CV data are very similar to those reported for OSPC-1a, suggesting that OSPC-1b and OSPC-0b store energy likewise through electrochemically and geometrically nonequivalent Li sites over a spectrum of interaction energies.^10,28−30^ We do not see the typical plateau associated with lithium intercalation in graphite and corresponding redox peaks; therefore, we infer that Li uptake occurs through an alternative mechanism.^30^ Heasman et al.^15^ rationalized the uptake mechanism and ICE for OSPC-1a through irreversible and reversible binding of lithium ions to the sp^3^ carbon node. This irreversible binding leads to a reduction in cell efficiency in the first cycle. Graphite exhibits a similar loss in efficiency at a similar current due to irreversible lithium ion uptake into the SEI, reported at 28%.^35^ Due to the similarities of the electrochemical characterization for OSPC-1b and OSPC-0b, we assign the same mechanism here.^10,14,15^ After 200 cycles, the discharge capacities for OSPC-1b and OSPC-0b were 502 and 440 mA h g^–1^, respectively.

We attributed these differences to the different polymerization route used in the one-pot synthesis compared to the six-step synthesis reported, as well as the narrower voltage window used in this work. As the different polymerization route results in different end groups that contain bromine and trimethyl silicon, which will be most prominent at the edges of the network, the surface chemistry could be altered, affecting the SEI composition. These end groups are residual from the synthesis and comprise less than 5% of the network.

Achieving high energy densities under a range of charging conditions is an important aspect of the lifetime of commercial batteries. Capacity fade due to structural degradation while fast charging as well as the ability to fast charge as a battery are prominent issues for commercial LIBs. The excellent long-term capacity retention as well as the absence of observable lithium dendrites under different electrochemical regimes is a stand-out feature of OSPC-1a.^10^

**Figure 6:**
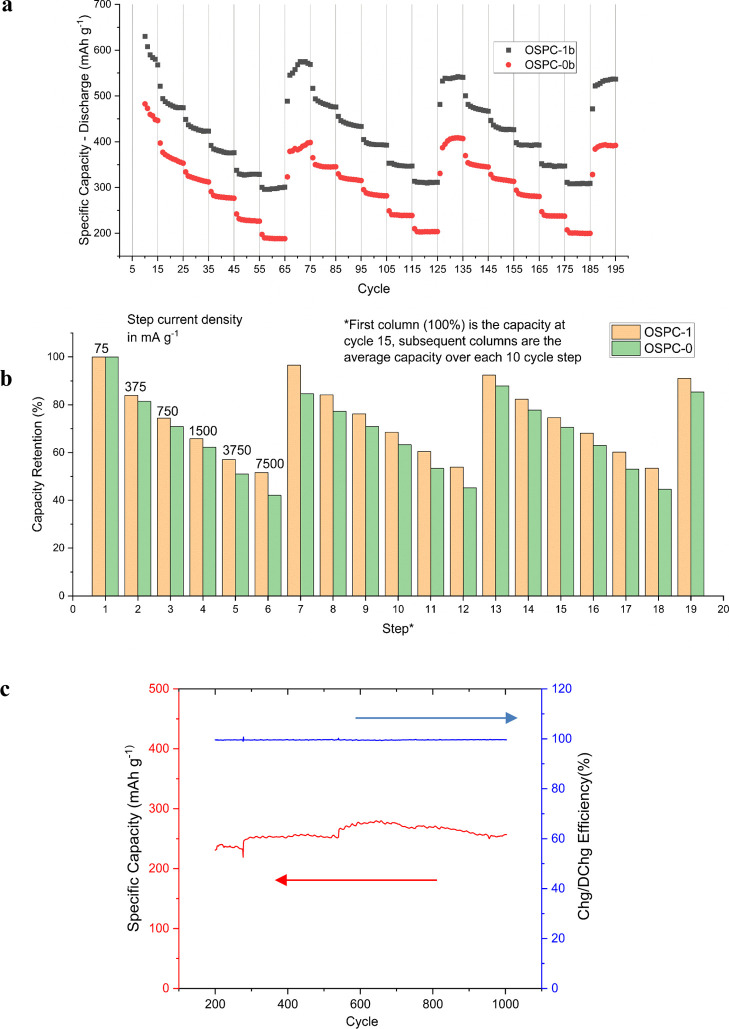
(a) Discharge capacity of OSPC-1b (black) and OSPC-0b (red) during the rate capability test. The cells were cycled 15 times at 75 mA g^–1^ for initial conditioning and then three times through a waterfall pattern of 10 cycles at 375, 750, 1500, 3750, 7500, and 75 mA g^–1^. (b) Capacity retention of OSPC-1b (orange) and OSPC-0b (green), relative to their initial capacity across the waterfall pattern at 75, 375, 750, 1500, 3750, 7500, and 75 mA g^–1^. (c) Long-term cycling performance of an OSPC-1b cell showing discharge capacity in the 0–2 V voltage window vs Li+/Li at a current density of 200 mA g^–1^ over cycles 2 to 1000.

For a battery system to be fast charging, high capacity must be observed with a current density of 1000 mA g^–1^ or above.^18^ A useful capacity observed at 7500 mA g^–1^ is considered to be exceptional with a previously reported state-of-the-art system, such as modified graphite and graphene nanocomposites, having capacities between 300 and 350 mA h g^–1^ after 10 cycles at 4000 mA g^–1^.^31^ To assess the fast-charging performance of the OSPC systems, we performed rate capability testing and compared the results to the graphite performance. We investigated the rate capability of OSPC-1b, OSPC-0b, and commercial graphite (for reference) at current densities from 75 to 7500 mA g^–1^, as shown in Figure 6a. OSPC-1b exhibited reversible capacities of 393, 347, and 309 mA h g^–1^ at current densities of 1500, 3750, and 7500 mA g^–1^, respectively. Figure 6b shows that OSPC-1b retained over half of its initial 577 mA h g^–1^ capacity measured at 75 mA g^–1^ at all current densities tested and recovered 94% of its initial capacity after the third cycle through the waterfall plot. This compares well with OSPC-1a, which reported a specific capacity of 356 mA h g^–1^ at a current density of 3000 mA g^–1^ and with fast charging optimized carbon-based LIB anode materials.^10,32,33^ OSPC-0b performed slightly less well than OSPC-1b, with reversible capacities of 284, 239, and 201 mA h g^–1^ at current densities of 1500, 3750, and 7500 mA g^–1^, respectively, compared to a capacity of 396 mA h g^–1^ at a current density of 75 mA g^–1^. The capacity retention percentages of OSPC-0b were lower than those of OSPC-1b at all capacities tested, with the difference increasing with increasing current density. Computational assessment of the diffusion of lithium ions in OSPC-0 and OSPC-1 networks suggested that the diffusion rate of lithium will be lower in OSPC-0 than in OSPC-1 due to the denser more rigid network and smaller pore sizes.^13^ The results here appear to back this prediction as we would expect that fast charging would be more challenging in a system with slower diffusion rates, as is observed for OSPC-0.

Long-cycling data (up to 1000 cycles) were collected for OSPC-1b, shown in Figure 5c, showing exceptional reversibility at a current of 200 mA g^–1^. This long-term resistance to capacity fade is consistent with electrochemical data for OSPC-1a,^10^ albeit slightly lower capacities were obtained for OSPC-1b than for OSPC-1a, which was previously reported in the literature, where a deficit of 200–250 mA h g^–1^ was observed after 100 cycles. The recently reported silicon OSPC analogue had a slightly higher capacity than OSPC-1a at around 900 mA h g^–1^ at 200 mA g^–1^ with similar long-term cycling performance, but the other silicon node networks reported along with it had capacities more in line with OSPC-0b. This is in line with the reported surface areas for the materials.^16^ The germanium OSPC analogue has capacities almost an order of magnitude higher than OSPC-1a, also with strong capacity retention that was tested to over 8000 cycles at high current density. However, the reaction procedure was modified to build the network as a thin film on copper foil, so it is not directly comparable to measurements on OSPC-1a and 1b powders.^16^

**Figure 7:**
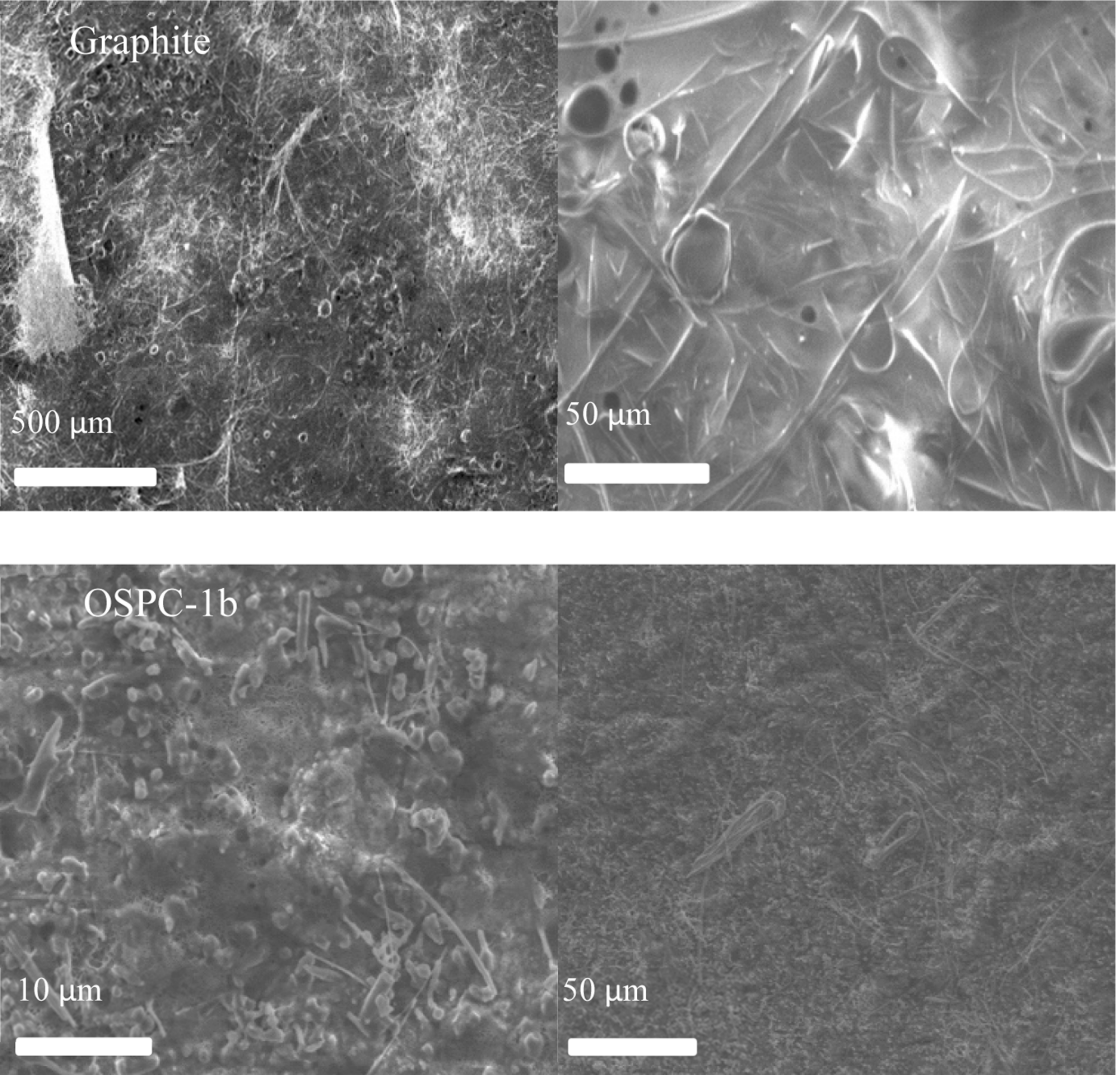
SEM images of OSPC-1b and graphite after overdischarging at 5000 mA h g^–1^ for 6 min.

A lithium plating experiment was carried out to induce the Li electrodeposition that can occur during overcharging to directly test OSPC-1’s resistance to Li plating in the absence of other variables.^16,34,36^ Dendrites from metallic lithium are deposited on the anode surface, reducing the ability to store charge in the material. These can further grow across the cathode, resulting in a short circuit and leading to potentially catastrophic failure of the battery. Figure 7 shows SEM images of the overdischarged electrodes. In the used graphite sample, we can see ball- or pebble-like features at a 50 μm scale, which can be seen across the surface at the 500 μm scale. In the OSPC-1b samples, these features are substantially smaller and less prevalent. For reference, pristine electrodes are shown in Figure S16. We attribute the presence of these ball- or pebble-like deposits to electrodeposited lithium metal in a globule or moss-type morphology. Mossy lithium is a precursor to dendrite growth, which we observe in the graphite cells so its absence on OSPC-1b suggests resistance to dendrite growth. To further probe the resilience of OSPC-1b electrodes, equivalent OSPC-1b and graphite cells were subjected to a stress test cycling pattern of 1000 cycles at 5000 mA g^–1^. After this, they were examined by XRD to capture a more bulk view of the electrode surface than is possible with SEM, and the results are shown in Figure S15.^37^ For the OSPC-1b anode, we see some graphitization after the stress test but no metallic lithium. For the graphite anode, we see severe degradation of the graphite lattice from intercalated lithium and the effect of repeated intercalation and deintercalation of lithium from the stress test’s conditions, as well as the presence of a lithium metal impurity phase.

## Conclusions

In conclusion, we have shown that the OSPC family of materials can be synthesized through a simple and fast one-pot method from commercially available reagents, giving larger yields of material with respect to OPSC-1a. Further, the resulting materials, OSPC-0b and OSPC-1b, show exceptional electrochemical properties, especially their fast-charging performance and outstanding resistance to degradation and lithium deposition. OSPC-0b has been electrochemically characterized for the first time showing good lithium-ion uptake properties. This opens a robust strategy for the design and targeting of OSPC materials with specific electrochemical properties. The existence of a more practical route creates new opportunities for exploration, including the performance with other metal-ion battery systems, and to further expand the family of OSPC materials.

## Materials and Methods

Bistrimethylsilyl acetylene (99%), cesium fluoride (99%), and DPS (99%) were obtained from Alfa Aesar. Bistrimethylsilyl butadiyne (98%) was obtained from both Alfa Aesar and Manchester Organics. Carbon tetrabromide (99%) and dichlorobenzene (99%) were both obtained from Sigma-Aldrich. *N*-Methyl-2-pyrrolidone was obtained from Fisher Scientific. All chemicals were used as purchased unless otherwise stated. Microwave vials were obtained from Biotage. Commercial graphite electrodes were obtained from MTI. The stainless-steel reactor and insert were obtained from BAOSHISHAN UK.

General procedure for the synthesis of OSPCs: Bis(trimethylsilyl)acetylene or bis(trimethylsilyl) diacetylene (2 equiv), carbon tetrabromide (1 equiv), CsF (5 equiv), and DPS (2.5 g per equiv) were combined in a container and sealed. The mixture was heated to 250 °C overnight and then cooled to 150 °C, and dichlorobenzene (5 mL per equiv) was added to loosen the crude product. The black precipitate was filtered out and suspended in NaOH solution (2 M) overnight. The black solid was collected by filtration and washed with water (25 mL) and then purified by Soxhlet extraction with methanol overnight. The product was then dried under vacuum overnight to give OSPC-1 or 0 as a black powder.

XPS analysis was performed on a Kratos Analytical Axis Supra using a monochromated Al Kα source. Solid-state NMR (ssNMR) was performed on a Bruker AVANCE III HD 700 Wb using a 3.2 mm probe. Raman spectra were obtained using the Raman InVia System (Renishaw plc, Wooton-Under edge U.K.) with a 532 nm laser with a power of 15 mW. SEM and EDX were performed on a JEOL JSM-7800F fitted with an X-Max50, large area 50 mm^2^ silicon drift detector (SDD) from Oxford Instruments. Polymer surface areas and pore size distributions were measured by nitrogen adsorption and desorption at 77.4 K using a Micrometrics 3-Flex adsorption analyzer.

The electrochemical performance of the studied materials was evaluated using stainless steel CR2032 coin cells (Tob New Energy) with Li metal as the reference and counter electrodes, 1 M LiPF_6_ in EC/DMC (1:1 w/w %) as the electrolyte, and a 17 mm Whatman micro glass fiber separator. The active material was mixed with carbon black (Super P) (99% Alfa Aesar) and polyvinylidene binder (PVDF Kynar, 99% Alfa Aesar) in a ratio of 5:4:1 at loadings of ca. 0.5 mg cm^–2^. GCD measurements were performed on a Neware battery cycler under the stated conditions. CV was carried out in the voltage window 0–3 vs Li^+^/Li at a scan rate of 10 mV s^–1^ performed on a potentiostat (VMP300, Biologic).

## Supporting Information Available

Instrumentation, materials, synthetic procedure, reaction conditions study, and full characterization data (PDF)

## References

[ref1] Wood C. D.; Tan B.; Trewin A.; Niu H.; Bradshaw D.; Rosseinsky M. J.; Khimyak Y. Z.; Campbell N. L.; Kirk R.; Stöckel E.; et al. Hydrogen Storage in Microporous Hypercrosslinked Organic Polymer Networks. Chem. Mater. 2007, 19 (8), 2034–2048. 10.1021/cm070356a.

[ref2] Jiang J.; Su F.; Trewin A.; Wood C. D.; Campbell N. L.; Niu H.; Dickinson C.; Ganin A. Y.; Rosseinsky M. J.; Khimyak Y. Z.; et al. Conjugated microporous poly (aryleneethynylene) networks. Angew. Chem., Int. Ed. 2007, 46 (45), 8574–8578. 10.1002/anie.200701595. 17899616

[ref3] Weber J.; Thomas A. Toward Stable Interfaces in Conjugated Polymers: Microporous Poly(p-phenylene) and Poly(phenyleneethynylene) Based on a Spirobifluorene Building Block. J. Am. Chem. Soc. 2008, 130 (20), 6334–6335. 10.1021/ja801691x. 18433126

[ref4] McKeown N. B.; Budd P. M. Polymers of intrinsic microporosity (PIMs): organic materials for membrane separations, heterogeneous catalysis and hydrogen storage. Chem. Soc. Rev. 2006, 35 (8), 675–683. 10.1039/b600349d. 16862268

[ref5] Kuhn P.; Antonietti M.; Thomas A. Porous, Covalent Triazine-Based Frameworks Prepared by Ionothermal Synthesis. Angew. Chem., Int. Ed. 2008, 47 (18), 3450–3453. 10.1002/anie.200705710. 18330878

[ref6] Liu A.; Mollart C.; Trewin A.; Fan X.; Lau C. H. Photo-Modulating CO2 Uptake of Hypercross-linked Polymers Upcycled from Polystyrene Waste. ChemSusChem 2023, 16 (10), e202300019 10.1002/cssc.202300019. 36772914

[ref7] Wang C.; Yan T.; Xing G.; Bailey S.; Lambert C.; Fayon P.; Trewin A.; Ben T. Electron and proton conducting framework organic salt single crystals. J. Solid State Chem. 2022, 308, 122903 10.1016/j.jssc.2022.122903.

[ref8] Fayon P.; Thomas J. M. H.; Trewin A. Structure and Properties of a Nanoporous Supercapacitor. J. Phys. Chem. C 2016, 120 (45), 25880–25891. 10.1021/acs.jpcc.6b08712.

[ref9] Kou Y.; Xu Y.; Guo Z.; Jiang D. Supercapacitive Energy Storage and Electric Power Supply Using an Aza-Fused π-Conjugated Microporous Framework. Angew. Chem., Int. Ed. 2011, 50 (37), 8753–8757. 10.1002/anie.201103493. 21842523

[ref10] Zhao Z.; Das S.; Xing G.; Fayon P.; Heasman P.; Jay M.; Bailey S.; Lambert C.; Yamada H.; Wakihara T.; et al. A 3D Organically Synthesized Porous Carbon Material for Lithium-Ion Batteries. Angew. Chem., Int. Ed. 2018, 57 (37), 11952–11956. 10.1002/anie.201805924. 29904996

[ref11] Frenck L.; Sethi G. K.; Maslyn J. A.; Balsara N. P. Factors That Control the Formation of Dendrites and Other Morphologies on Lithium Metal Anodes. Front. Energy Res. 2019, 7, 115 10.3389/fenrg.2019.00115.

[ref12] Son Y.; Lee T.; Wen B.; Ma J.; Jo C.; Cho Y.-G.; Boies A.; Cho J.; De Volder M. High energy density anodes using hybrid Li intercalation and plating mechanisms on natural graphite. Energy Environ. Sci. 2020, 13 (10), 3723–3731. 10.1039/D0EE02230F.

[ref13] Wu J.; Rui X.; Wang C.; Pei W.-B.; Lau R.; Yan Q.; Zhang Q. Nanostructured Conjugated Ladder Polymers for Stable and Fast Lithium Storage Anodes with High-Capacity. Adv. Energy Mater. 2015, 5 (9), 1402189 10.1002/aenm.201402189.

[ref14] Heasman P.; Trewin A. Uptake and Diffusion of Ions in Organically Synthesized Porous Carbon for Battery Anode Applications. J. Phys. Chem. C 2019, 123 (42), 25603–25610. 10.1021/acs.jpcc.9b07878.

[ref15] Heasman P.; Varley E.; Trewin A. Lithium-Ion Uptake and Diffusion in a Family of Organically Synthesized Porous Carbon. Energy Fuels 2022, 36 (12), 6560–6568. 10.1021/acs.energyfuels.2c00646.

[ref16] Dong C.; Chu J.; Cao L.; Cui F.; Liang S.; Zhang X.; Tao X.; Wang H.-g.; Zhu G. A Three-Dimensional Silicon-Diacetylene Porous Organic Radical Polymer. CCS Chem. 2023, 5 (3), 607–615. 10.31635/ccschem.022.202202351.

[ref17] Yang Z.; Ren X.; Song Y.; Li X.; Zhang C.; Hu X.; He J.; Li J.; Huang C. Germanium-Carbdiyne: A 3D Well-Defined sp-Hybridized Carbon-Based Material with Superhigh Li Storage Property. Energy Environ. Mater. 2023, 6 (1), 12269 10.1002/eem2.12269.

[ref18] Wang J.; Fu X.; Yan N.; Zhang Y. Molecular Design of 3D Porous Carbon Framework via One-Step Organic Synthesis. ChemSusChem 2021, 14 (18), 3806–3809. 10.1002/cssc.202101262. 34263532

[ref19] Mollart C.; Trewin A. Conjugated microporous polymer frameworks for sustainable energy materials – elucidating the influence of solvents on the porosity properties for future design principles. J. Mater. Chem. A 2024, 12 (7), 4159–4168. 10.1039/D3TA04866G.

[ref20] Mollart C.; Trewin A. Rationalising the influence of solvent choice on the porosity of conjugated microporous polymers. Phys. Chem. Chem. Phys. 2020, 22 (38), 21642–21645. 10.1039/D0CP03539D. 32968748

[ref21] Dawson R.; Laybourn A.; Clowes R.; Khimyak Y. Z.; Adams D. J.; Cooper A. I. Functionalized Conjugated Microporous Polymers. Macromolecules 2009, 42 (22), 8809–8816. 10.1021/ma901801s.

[ref22] Jiang J.-X.; Su F.; Trewin A.; Wood C. D.; Campbell N. L.; Niu H.; Dickinson C.; Ganin A. Y.; Rosseinsky M. J.; Khimyak Y. Z.; et al. Conjugated Microporous Poly(aryleneethynylene) Networks. Angew. Chem., Int. Ed. 2007, 46 (45), 8574–8578. 10.1002/anie.200701595. 17899616

[ref23] Dawson R.; Cooper A. I.; Adams D. J. Nanoporous organic polymer networks. Prog. Polym. Sci. 2012, 37 (4), 530–563. 10.1016/j.progpolymsci.2011.09.002.

[ref24] Ren S.; Bojdys M. J.; Dawson R.; Laybourn A.; Khimyak Y. Z.; Adams D. J.; Cooper A. I. Porous, Fluorescent, Covalent Triazine-Based Frameworks Via Room-Temperature and Microwave-Assisted Synthesis. Adv. Mater. 2012, 24 (17), 2357–2361. 10.1002/adma.201200751. 22488602

[ref25] Katekomol P.; Roeser J.; Bojdys M.; Weber J.; Thomas A. Covalent Triazine Frameworks Prepared from 1,3,5-Tricyanobenzene. Chem. Mater. 2013, 25 (9), 1542–1548. 10.1021/cm303751n.

[ref26] Dubale A. A.; Su W.-N.; Tamirat A. G.; Pan C.-J.; Aragaw B. A.; Chen H.-M.; Chen C.-H.; Hwang B.-J. The synergetic effect of graphene on Cu2O nanowire arrays as a highly efficient hydrogen evolution photocathode in water splitting. J. Mater. Chem. A 2014, 2 (43), 18383–18397. 10.1039/C4TA03464C.

[ref27] Castiglioni C.; Tommasini M.; Zerbi G.; Tommasini M.; Zerbi G. Raman spectroscopy of polyconjugated molecules and materials: confinement effect in one and two dimensions. Philos. Trans. R. Soc., A 2004, 362 (1824), 2425–2459. 10.1098/rsta.2004.1448. 15482986

[ref28] Li X.; Geng D.; Zhang Y.; Meng X.; Li R.; Sun X. Superior cycle stability of nitrogen-doped graphene nanosheets as anodes for lithium ion batteries. Electrochem. Commun. 2011, 13 (8), 822–825. 10.1016/j.elecom.2011.05.012.

[ref29] Yang Z.-h.; Wu H.-q. Electrochemical intercalation of lithium into fullerene soot. Mater. Lett. 2001, 50 (2–3), 108–114. 10.1016/S0167-577X(00)00425-0.

[ref30] Galinski M.; Acznik I. Study of a graphene-like anode material in N-methyl-N-propylpyrrolidinium bis(trifluoromethanesulfonyl)imide ionic liquid for Li-ion batteries. J. Power Sources 2012, 216, 5–10. 10.1016/j.jpowsour.2012.05.039.

[ref31] Li L.; Zhang D.; Deng J.; Gou Y.; Fang J.; Cui H.; Zhao Y.; Cao M. Carbon-based materials for fast charging lithium-ion batteries. Carbon 2021, 183, 721–734. 10.1016/j.carbon.2021.07.053.

[ref32] Zhu G.-L.; Zhao C.-Z.; Huang J.-Q.; He C.; Zhang J.; Chen S.; Xu L.; Yuan H.; Zhang Q. Fast Charging Lithium Batteries: Recent Progress and Future Prospects. Small 2019, 15 (15), 1805389 10.1002/smll.201805389. 30869836

[ref33] Zhang D.; Tan C.; Zhang W.; Pan W.; Wang Q.; Li L. Expanded Graphite-Based Materials for Supercapacitors: A Review. Molecules 2022, 27 (3), 716 10.3390/molecules27030716. 35163981 PMC8839398

[ref34] Pang B.; Yang T.; Wu Z.; Li Z.; Jin Z.; Zhang W.; Xia Y.; Huang H.; He X.; Gan Y. X.5-Based All-Solid-State Battery with a Silver Nanoparticle-Modified Graphite Anode for Improved Resistance to Overcharging and Increased Energy Density. ACS Appl. Mater. Interfaces 2024, 16 (16), 20510–20519. 10.1021/acsami.4c01172. 38623904

[ref35] Libich J.; Máca J.; Vondrák J.; Čech O.; Sedlaříková M. Irreversible capacity and rate-capability properties of lithium-ion negative electrode based on natural graphite. J. Energy Storage 2017, 14, 383–390. 10.1016/j.est.2017.03.017.

[ref36] Lu W.; López C. M.; Liu N.; Vaughey J. T.; Jansen A.; Dennis D. Overcharge effect on morphology and structure of carbon electrodes for lithium-ion batteries. J. Electrochem. Soc. 2012, 159 (5), A566–A570. 10.1149/2.jes035205.

[ref37] Borkiewicz O. J.; Wiaderek K. M.; Chupas P. J.; Chapman K. W. Best practices for operando battery experiments: influences of X-ray experiment design on observed electrochemical reactivity. J. Phys. Chem. Lett. 2015, 6 (11), 2081–2085. 10.1021/acs.jpclett.5b00891. 26266506

