# Supplementary material for: Facile Synthesis of Organically Synthesized Porous Carbon Using a Commercially Available Route with Exceptional Electrochemical Performance
Source: ACS Appl Mater Interfaces. 2024 Aug 26;16(36):47631–8. doi: 10.1021/acsami.4c09710 (PMC11403544; doi:10.1021/acsami.4c09710)
Supplement: Supplementary file 1 — am4c09710_si_001.pdf [file am4c09710_si_001.pdf]

Supporting Information

Facile synthesis of organically synthesised porous carbon using a commercially available route with exceptional electrochemical performance

Adam Rowling,[a] Julien Doulcet,[a] Robert Dawson,[b] Nuria Tapia-Ruiz,[c]* and Abbie Trewin[a]*

[a] Department of Chemistry Lancaster University Bailrigg, Lancaster, LA1 4YB, UK E-mail: a.trewin@lancaster.ac.uk
[b] Department of Chemistry, Dainton Building, 13 Brook Hill, Sheffield, S3 7HF, Email r.dawson@sheffield.ac.uk
[c] Department of Chemistry, Molecular Sciences Research Hub, White City Campus, Imperial College London, London, W12 0BZ. Email: n.tapia-ruiz@imperial.ac.uk

Contents

1. Instruments
1.1 X-ray photoelectron spectroscopy (XPS)
1.2 Solid state NMR
1.3 Raman analysis
1.4 Scanning electron microscopy (SEM) and energy dispersive X-ray spectroscopy (EDX)
1.5 Surface area analysis
1.6 CHNS analysis
1.7 Electrochemical performance in Li-ion batteries
1.8 X-ray Diffraction
2. Materials
3. Synthetic procedures
3.1 General procedure 1: Synthesis of OSPC-0b
3.2 General procedure 2: Synthesis of OSPC-1b
3.3 General procedure 3: Large scale synthesis of OSPC-1b
4. Study of reaction conditions for OSPC synthesis.
4.1 Synthetic route for OSPC-1a
4.2 Solvent screening for OSPC-0b and OSPC-1b
4.3 Large scale reaction in round bottom flask and a stainless-steel reactor
4.4 Percentage yield calculations
4.5 Cost breakdown
5. Solid state NMR of OSPC-1b and OSPC-0b
6. Raman Spectroscopy of OSPC-1b and OSPC-0b
7. Computational Raman Spectroscopy of OSPC-0b
8. Surface area analysis
9. CHNS elemental analysis
10. X-ray Photoelectron Spectroscopy
11. SEM and EDX
12. Electrochemical analysis
13. SEM Over-discharge investigation

14. XRD stress test investigation
15. References

1. Instruments

1.1 X-ray photoelectron spectroscopy (XPS)

XPS analysis was performed on a Kratos Analytical Axis Supra using a monochromated Al Kα source, which investigated the wide spectrum, the C 1s region and the O 1s region. Six sites were analysed on each sample, one site on the OSPC-1b sample showed evidence of decomposition and was not included in the analysis. Data analysis and peak fitting was performed on CasaXPS.

1.2 Solid state NMR

Solid state NMR (ssNMR) was performed on a Bruker AVANCE III HD 700Wb using a 3.2 mm probe. The results were obtained through the Hahn echo direct excitation of ^{13}C. The MAS frequency was 16 kHz and the D1 was 300s for OSPC-1b and 60s for OSPC-0b.

Additional ssNMR was performed on a Bruker AVANCE III HD 400Wb using a 2.6 mm probe. The results were obtained through the Hahn echo direct excitation of ^{13}C. The MAS frequency was 25 kHz and the D1 was 60s.

1.3 Raman analysis

Raman spectra were obtained using the Raman InVia System (Renishaw plc, Wooton-Under edge U.K.) with a 532 nm laser with a power of 15mW.
OSPC-0b: Six individual static spectra centred at 1200 cm^{-1} were obtained using an exposure time of 2 seconds at 100% laser power for 10 accumulations. An additional extended spectrum was obtained using a 10 second exposure time at 100% laser power for 1 accumulation.

OSPC-1b: Six individual static spectra centred at 1200 cm^{-1} were obtained using an exposure time of 2 seconds at 1% laser power for 10 accumulations. An additional extended spectrum was obtained using a 10 second exposure time at 1% laser power for 1 accumulation.
300s.

1.4 Scanning electron microscopy (SEM) and energy dispersive X-ray spectroscopy (EDX)

SEM and EDX were performed on a JEOL JSM-7800F fitted with a X-Max50, large area 50 mm^2 Silicon Drift Detector (SDD) from Oxford Instruments. Images were captured at between 100x and 30000x magnification at 2.00 kV acceleration. Powders were dropped onto carbon tabs ((G3348N, Agar Scientific) for this measurement.

1.5 Surface area analysis

Polymer surface areas and pore size distributions were measured by nitrogen adsorption and desorption at 77.4 K using a Micrometrics 3-Flex adsorption analyser over a relative pressure range (P / P_0) of 0.01 to 0.25. Before the analysis, the materials were outgassed under a primary vacuum at 100 °C for 24 hours.

1.6 CHNS analysis

CHNS analysis was performed on a Vario MICRO cube using 2 mg samples

1.7 Electrochemical performance in Li-ion batteries

The electrochemical performance of the studied materials was evaluated using stainless steel CR2032 coin cells (Tob New Energy) with Li metal as the reference and counter electrode, 1M $LiPF_6$ in EC/DMC (1:1 w/w%) as the electrolyte, and a 17 mm Whatman micro class fibre separator.

Assembly was performed in an argon filled glovebox (MBraun) with O_2 and $H_2O \leq 0.1$ ppm, where the active material was mixed with carbon black (Super P) (99 % Alfa Aesar) and polyvinylidene binder (PVDF Kynar, 99 % Alfa Aesar) in a ratio of 5:4:1 at loadings of circa 0.5 mg cm^{-2}.

The mixture was cast onto copper foil (99.98%, 12 µm, Tob New Energy) acting as current collector, punched into 0.785 mm^2 electrodes, and pressed under 5 tonnes with a hydraulic press. Galvanostatic charge/discharge measurements were performed on Neware battery cycler under the stated conditions.

Cyclic voltammetry (CV) was carried out in the voltage window 0-3 vs. Li^+/Li at a scan rate of 10 mV s^{-1} performed on a potentiostat (VMP300, Biologic).

Over discharge experiments were performed by discharging the OSPC-1b and graphite cells from their respective open circuit voltages to 0.005V at 20 mA g^{-1} and then held at a discharge current of 5000 mA g^{-1} for 6 minutes with no lower voltage window. The cells were then disassembled and mounted on SEM stubs without washing. This preserved any dendrites on the surface but resulted in residual separator fibres visible on the SEM images.

The stress test cycling pattern consisted of 1000 cycles at 5000 mA g^{-1}. The cells were then disassembled and transferred under inert conditions to a separate glovebox containing the XRD machine. The samples were prepared on the aluminium sample holder under an inert atmosphere.

1.8 X-ray Diffraction

Powder X-ray diffraction (XRD) data were collected on a Rigaku MiniFlex600 at room temperature in Bragg-Brentano geometry with a 0.6 kW Cu-source generator (K_α = 1.5406 Å) and a D/teX Ultra detector. The powdered samples were packed on a flat circular glass holder and scanned from 5° to 90° 2θ.

2. Materials

Bistrimethylsilyl acetylene (99%), cesium fluoride (99%), and diphenyl sulfone (99%) were obtained from Alfa Aesar. Bistrimethylsilyl butadiyne (98%) was obtained from both Alfa Aesar and Manchester Organics. Carbon tetrabromide (99%) and dichlorobenzene (99%) were both obtained from Sigma-Aldrich. N-Methyl-2-pyrrolidone was obtained from Fisher Scientific. All chemicals were used as purchased unless otherwise stated. Microwave vials were obtained from Biotage. Commercial graphite electrodes were obtained from MTI. The stainless-steel reactor and insert were obtained from BAOSHISHAN UK

3. Synthetic procedures

3.1 General Procedure 1: Synthesis of OSPC-0b (Method B)

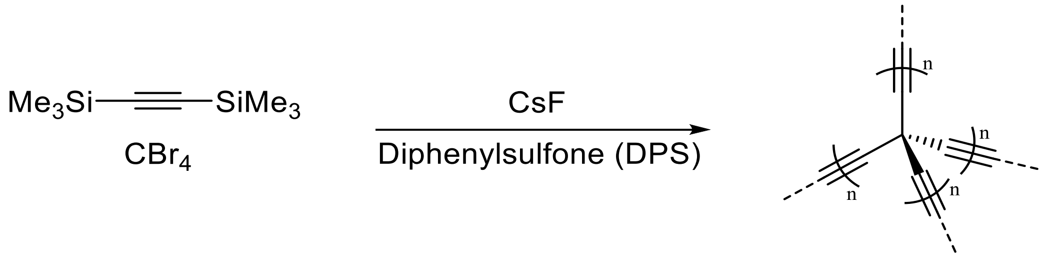

CsF (0.75 g, 5 mmol) was added a 20-mL microwave vial, and was dried using a heat gun under reduced pressure (600 °C, 5 mBar, 5 min). After cooling to room temperature, bis(trimethylsilyl)acetylene (0.34 g, 2 mmol), carbon tetrabromide (0.33 g, 1 mmol) and diphenyl sulfone (2.5 g) were added to the microwave vial (exposed to air). The microwave vial was capped, and the mixture was heated at 250 °C overnight. Then, the rection mixture was cooled to 150 °C and dichlorobenzene (5 mL) was added. The black precipitate was filtered out and was suspended in NaOH aq. solution (50 mL, 2M) overnight. The black solid was then collected by filtration and rinsed with H_2O (25 mL). The resultant solid underwent further purification using Soxhlet extraction with methanol overnight and the product was then dried under high vacuum overnight to give OSPC-0b (50 - 100 mg) as a black powder.

3.2 General procedure 2: Synthesis of OSPC-1b (Method B)

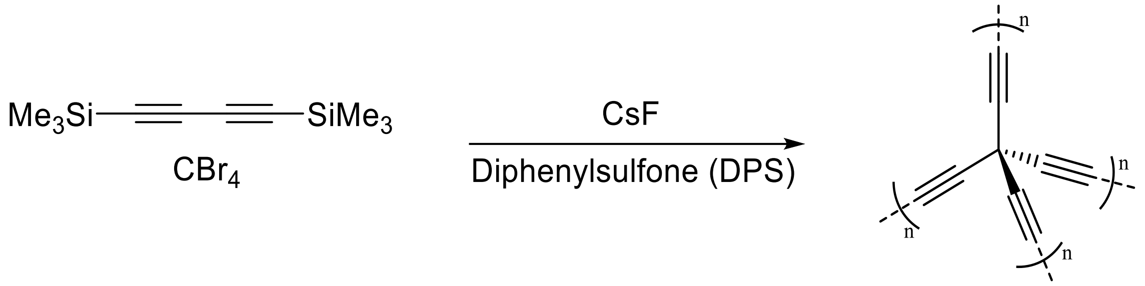

CsF (0.75 g, 5 mmol) was added a 20-mL microwave vial, and was dried using a heat gun under reduced pressure (600 °C, 5 mBar, 5 min). After cooling to room temperature, 1,4-bis(trimethylsilyl)-1,3-butadiyne (0.40 g, 2 mmol), carbon tetrabromide (0.33 g, 1 mmol) and diphenyl sulfone (2.5 g) were added to the microwave vial (exposed to air).. The microwave vial was capped, and the mixture was heated at 250 °C overnight. Then, the rection mixture was cooled to 150 °C and dichlorobenzene (5 mL) was added. The black precipitate was filtered out and was suspended in NaOH aq. solution (50 mL, 2M) overnight. The black solid was then collected by filtration and rinsed with H_2O (25 mL). The resultant solid underwent further purification using Soxhlet extraction with methanol overnight and the product was then dried under high vacuum overnight to give OSPC-1b (100 - 150 mg) as a black powder

3.3 General procedure 3: Large scale synthesis of OSPC-1b (Method B)

Then, 1,4-bis(trimethylsilyl)-1,3-butadiyne (3.11 g, 16 mmol), carbon tetrabromide (2.64 g, 8 mmol), dried CsF (6 g, 40 mmol) and diphenyl sulfone (20 g) were added to a 100-mL stainless steel reactor (equipped with a PTFE insert) that had been heated to 150 ˚C. The reactor was sealed and heated to 200 ˚C overnight and then allowed to cool to room temperature. Acetone (approx. 100 mL) was added to loosen the crude product (by dissolving diphenylsulfone) and the black precipitate was filtered out. The resultant solid underwent further purification using Soxhlet extraction with methanol overnight and the product was then dried under vacuum overnight to give OSPC-1b (1.9 g) as a black powder.

4. Study of reaction conditions for OSPC synthesis.

4.1 Synthetic route for OSPC-1a synthesis (Method A)

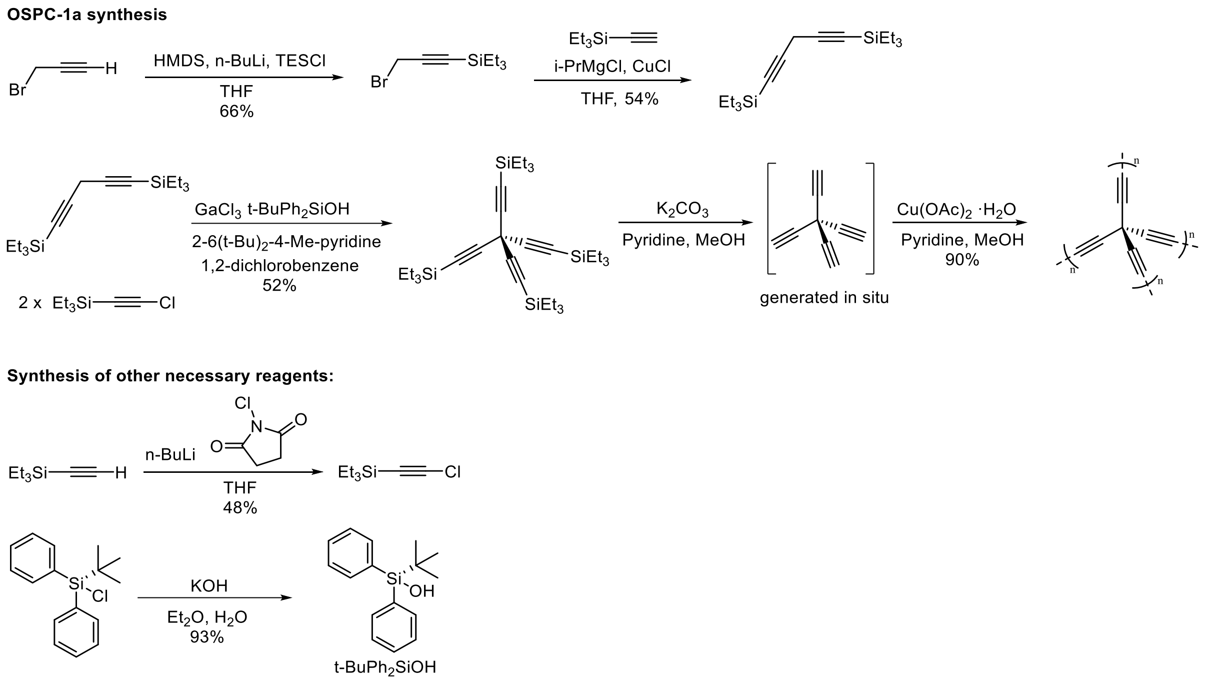

Figure S1: The synthetic procedure (Method A) used in the original synthesis of OSPC-1 (OSPC-1a).[1]

4.2 Solvent screening for OSPC-0b and OSPC-1b synthesis

Using general procedure 1, Mesitylene, diglyme, NMP, and 1,2-dichlorobenzene were investigated as alternative solvents to DPS in the synthesis of OSPC-0b. All reactions were run on a 1 mmol scale at 150 ˚C under otherwise identical conditions. DPS yielded significantly more material that the other solvents (Table S1), so was used for further synthetic work. It should be noted that discussing and comparing yields in the context of the synthetic method and variations on it is problematic. In the multistep A method if only 70% of the monomers was incorporated into the polymer network, the resulting network would largely resemble the theoretical structure, with the only significant deviations occurring at the end groups. However, with the one-pot method there is a potential for structural deviation around every sp^3 node. Furthermore, as the bromine end groups are greater in mass than the struts it is possible for a less complete network to weigh more than a more complete one. Therefore, calculating the percentage yield based on a theoretically perfect structure often gives yields above 100%. Alternatively, the yields could be calculated from the theoretical structure with the most mass. However, in both cases it would not be possible to determine if the deviation from the expected mass is due to the size of the network or to the amount of unreacted end groups remaining.
For this reason, we have not attempted to calculate a percentage yield and have instead reported the masses produced as is. This is not without issue, especially when discussing solvent screening. Solvent choice has been shown to affect the structure of microporous polymers.[2, 3]

Therefore, even if one solvent results in a network that has more mass than another, it may not be a “better” solvent if the additional mass is from end groups rather than a larger network. We did not consider this in our investigation, as our priority was obtaining a sufficient mass of sample for further analysis. However, a more detailed investigation of solvent effects, potentially from a computational angle could clarify this.

Table S1: Solvent screening for the synthesis of OSPC-0b according to general procedure 1 (reactions ran at 150 ˚C instead of 250 ˚C).

Solvent	Mass of OSPC-0b obtained
Mesitylene	9.7 mg
1,2-Dichlorobenzene	14.3 mg
NMP	12.6 mg
Diglyme	12.9 mg
DPS	34.4 mg

4.3 Large scale reaction in round bottom flask and a stainless-steel reactor

Our initial synthesis was performed in microwave vials, which limited the synthetic scale that could be used. An alternative set up that used a 2-necked flask, and a continuous flow of inert gas was investigated for the synthesis of OSPC-1b. When compared to an identically prepared sample made in a vial it yielded 71 mg of product in comparison to 154 mg. Scaling up the reaction by 150% and doubling the solvent slightly improved the relative yield to 121 mg, but it was still proportionally lower than the sealed vial. We felt that since the amount of material produced differed from the method using the microwave vial, the properties of OSPC-1b produced that way could differ too; the method using a two-necked RBF and a flow of nitrogen was therefore not further investigated.

We subsequently procured a basic 100 mL stainless-steel reactor with a PTFE insert. The reactor can be sealed but lacks the functionality to be purged with inert gas. To minimize the exposure of the reaction contents to water we pre-heated the reactor before adding the reactants. We ran the reaction at 4x the microwave vial scale which yielded 0.84 g of OSPC-1, and at 8x the original scale the scale which yielded 1.9 g of OSPC-1, a slightly greater than proportional increase. This suggests that the reaction is not as air/moisture sensitive as we had initially anticipated, and that it would respond positively to further increases in scale.

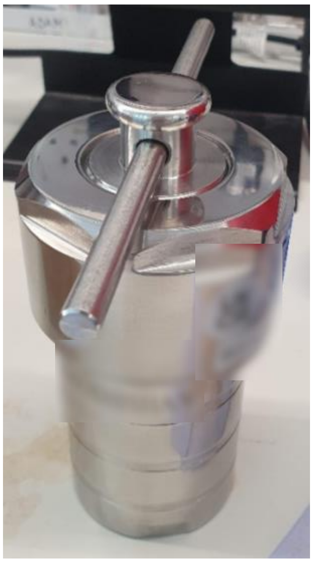

Figure S2: The reactor used for larger scale synthesis of OSPC-1b and 0b. Labels blurred.

4.4 Percentage yield calculations

Method A gives an overall yield of 17% for OSPC-1a over its longest linear sequence of steps and method B gives a 220% yield for OSPC-1b and a 130% yield OSPC-0b. Yields over 100% are common for these types of material due to the presence of end groups, trapped solvent, and absorbents from air including water, CO_2, and N_2. To further explore the yield and attempt to give an 'OSPC-only' yield, we have the determined further yields based on XPS, EDX, and CHNS data, see Figure S3 and below for an example of the calculation for the yield determined from XPS data. We believe that the XPS is more accurate than EDX and CHNS, but being a surface sensitive technique that this represents a lower limit of the yield.

OSPC-1a synthesis

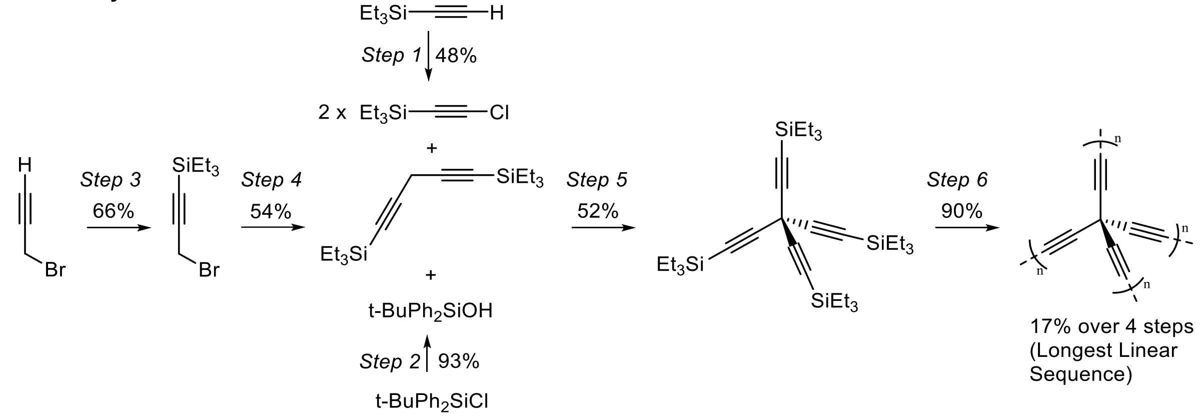

OSPC-1b synthesis

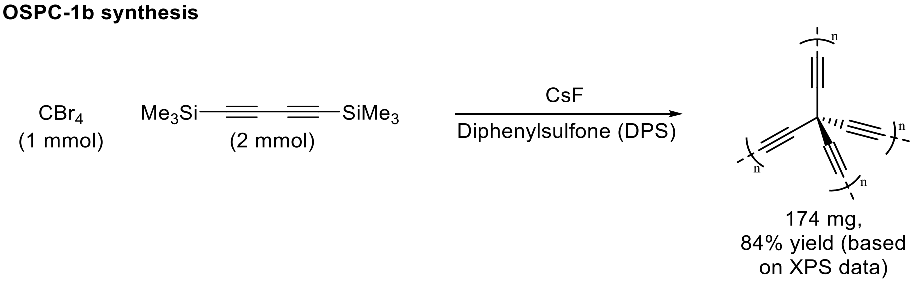

Description	Yield (%)	Problem
By mass OSPC-1b<br>By mass OSPC-0b	161<br>132	Does not take into account end groups, solvent, and absorbents
Adjusted with XPS OSPC-1b<br>Adjusted with XPS OSPC-0b	84<br>101	Surface technique only
Adjusted with EDX OSPC-1b<br>Adjusted with EDX OSPC-0b	46<br>86	Surface technique only
Adjusted with CHNS OSPC-1b<br>Adjusted with CHNS OSPC-0b	90<br>70	Does not take into account end groups, solvent, and absorbents

Figure S3: Yields for the synthesis of OSPC-1a (method A) and OSPC-1b (method B) and table of yields with adjusted values.

Calculation of the yield for OSPC-1b based on XPS results:

The OSPC-1b sample used for XPS analysis was obtained from a 1 mmol scale batch synthesis, giving 174 mg of OSPC-1b. If only the relevant carbon atoms were incorporated into the resulting network the theoretical yield (for a 1 mmol scale reaction) would be 108 mg. This gives a by-mass yield of 161%.

Considering the abundance of atoms observed in the wide XPS data (see table S7), we assume that all Si atoms observed are part of a $SiMe_3$ group, and therefore we remove an equivalent ratio of $(CH_3)_3$ from the mass.

This gives an adjusted yield of 84% for OSPC-1b. Additionally, XPS is a surface technique and a greater prevalence of end groups at the surface is expected, therefore this calculation may overestimate the abundance of end groups. We therefore propose this figure as a lower bound for the yield. This highlights the difficulties of discussing yields in the context of network materials, as described in section S4.2.

Calculation of the yield for OSPC-0b based on XPS results:

The OSPC-0b sample used for XPS analysis was obtained from a 1 mmol scale batch synthesis (as described in section S3.2), giving 79 mg of OSPC-0b. If only the relevant carbon atoms were incorporated into the resulting network the theoretical yield (for a 1 mmol scale reaction) would be 60 mg. This gives a by-mass yield of 132%.

Considering the abundance of atoms observed in the wide XPS data (see table S8), we assume that all Si atoms observed are part of a $SiMe_3$ group, and therefore we remove an equivalent ratio of $(CH_3)_3$ from the mass. This gives an adjusted yield of 101% for OSPC-0b.

4.5 Cost breakdown

Given the variability and location dependence of chemical prices, it is difficult to obtain a precise value for the cost delta between methods A and B. Here, we have attempted to provide a lower bound for this value by compiling the lowest price per gramme for the key structural reagents and excluding the cost of solvents, consumables, and energy. Any reagent that costs less than 0.01 GBP per g has been excluded. All prices were taken from Sigma-Aldrich, aside from Gallium trichloride which was unavailable so was taken from Fisher Scientific UK. All prices are accurate to 01/05/2024.

**Synthesis of 3-(triethylsilyl)propargyl bromide**	
Reagent	Price (£ GBP)
Hexamethyldisilazane 2.87 mL	0.525
n-BuLi in hexane 8.18 mL	1.432
Propargyl bromide 1.49g	0.98

➔ **3-(triethylsilyl)propargyl bromide 1.92g**	**£1.592 per g**
**Synthesis of 1,5-Bis(triethylsilyl)penta-1,4-diyne**	
MeMgBr in THF 16.5 mL	4.29
Triethylsilyl acetylene 3.6 mL	36.792
3-(triethylsilyl)propargyl bromide 3.31 g	5.06
➔ **1,5-Bis(triethylsilyl)penta-1,4-diyne 2.16g**	**£21.36 per g**
**Synthesis of (chloroethynyl) triethylsilane**	
Triethylsilyl acetylene 1.1 mL	23.24
n-BuLi in hexane 6.9 mL	1.208
N-Chlorosuccinimide 1.63 g	0.136
➔ **(Chloroethynyl) triethylsilane 0.84 g**	**29.27 per g**
**Synthesis of Triethylsilyl protected tetraethynylmethane**	
Gallium trichloride 0.176 g	0.635
2,6-di(tert-buty)-4-methylpyridine 0.2 g	1.176
1,5-Bis(triethylsilyl)penta-1,4-diyne 0.15 g	3.204
(chloroethynyl)triethylsilane 0.35 g	10.24
➔ **TES protected monomer**	**101.73 per g**
**Synthesis of OSPC-1a**	
TES protected monomer 0.105 g	10.68
Copper acetate 0.225 g	0.369
➔ **OSPC-1a 17 mg**	**649.94 per g**

**Synthesis of OSPC-1b**	
Bistrimethylsilyl butadiyne 3.11g	44.03
Carbon tetrabromide 2.64 g	0.84
Caesium Fluoride 6g	7.08
➔ **OSPC-1b 1.904 g**	**27.284 per g**

5. Solid state NMR of OSPC-1b and OSPC-0b

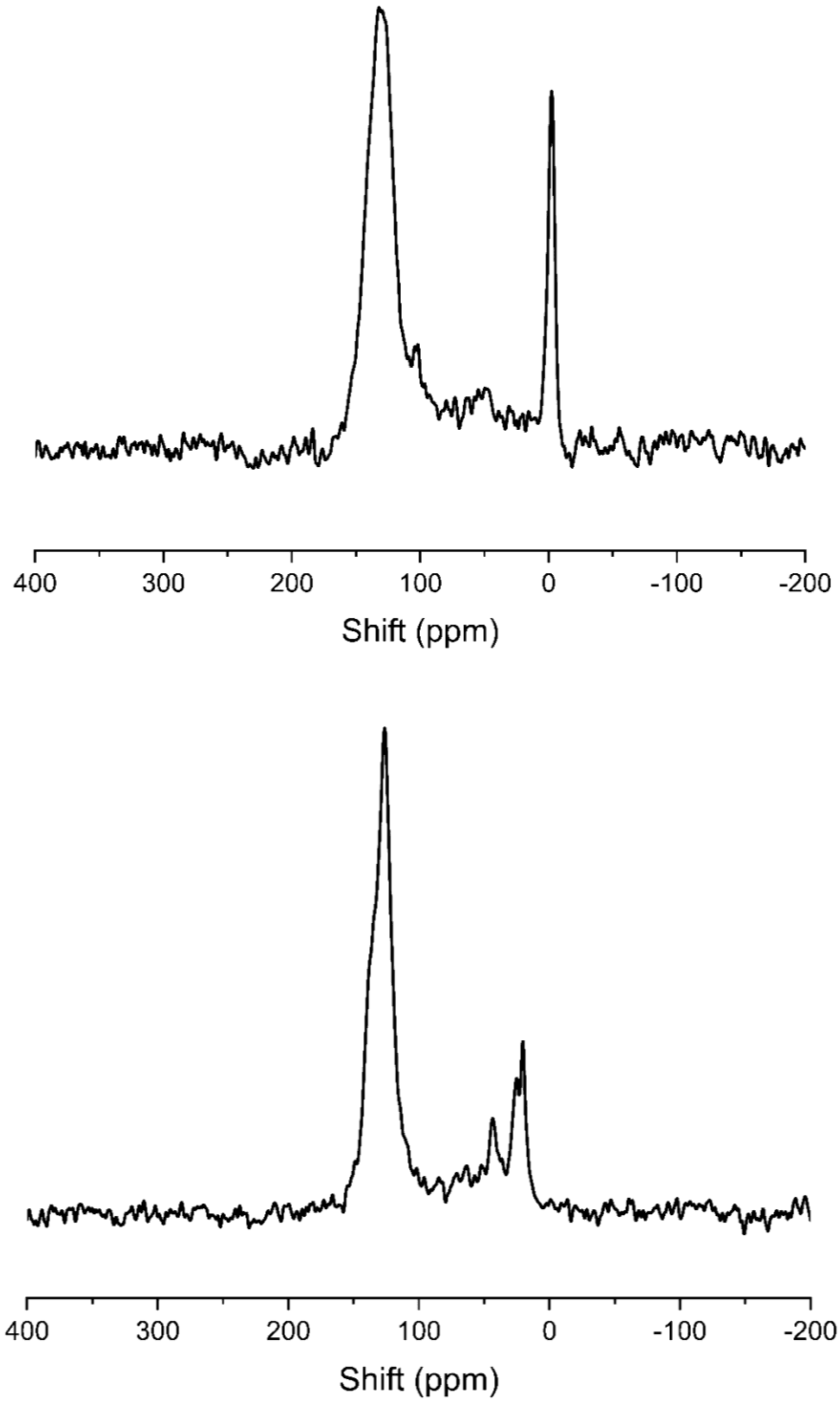

Figure S3: Solid state NMR spectra of OSPC-1b (top) and OSPC-0b (bottom) obtained at a high MAS speed of 25 kHz (compared to 16 kHz used to obtain the NMR spectra shown in Figure 3 (b)). In order to increase the spinning speed a smaller mass of sample had to be used with a lower resolution spectrometer.

6. Raman Spectroscopy of OSPC-1b and OSPC-0b

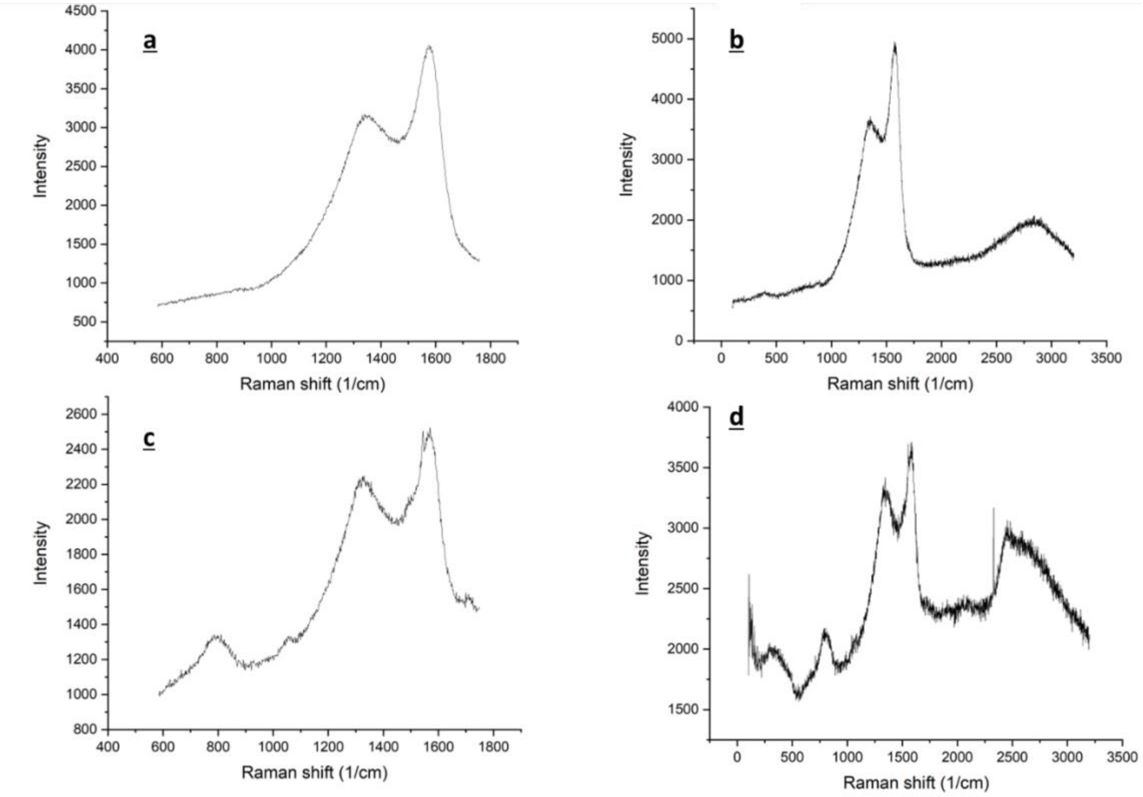

Figure S4: Raman spectra of OSPC-1b (a – focused, b – wide) and OSPC-0b (c – focused, d – wide).

7. Computational Raman Spectroscopy of OSPC-0b

The observed Raman spectra of OSPC-0b contains a peak at 800 cm^{-1} that is not observed in the spectra of OSPC-1b. In the absence of literature data to compare with, Raman spectra were calculated for fragments of OSPC-0 with gaussian basis sets of increasing complexity according to a previously published method.[1] Peaks resulting from vibrations that would not occur in an extended amorphous OSPC-0 structure were excluded. We confirmed that the two largest peaks at 1358 cm^{-1} and 1590 cm^{-1} were from the *sp* carbons, which agrees with previous gaussian analysis of OSPC-1. We believe the most likely source of the peak at 800 cm^{-1} is asymmetric stretching of the sp^3 bonds between the node carbons and the struts. The intensity of these stretches are significantly lower than those associated with the *sp* carbons. As OSPC-0 has a significantly greater density of nodal carbons compared to OSPC-0, we can see this stretch in OSPC-0, but not in OSPC-1.

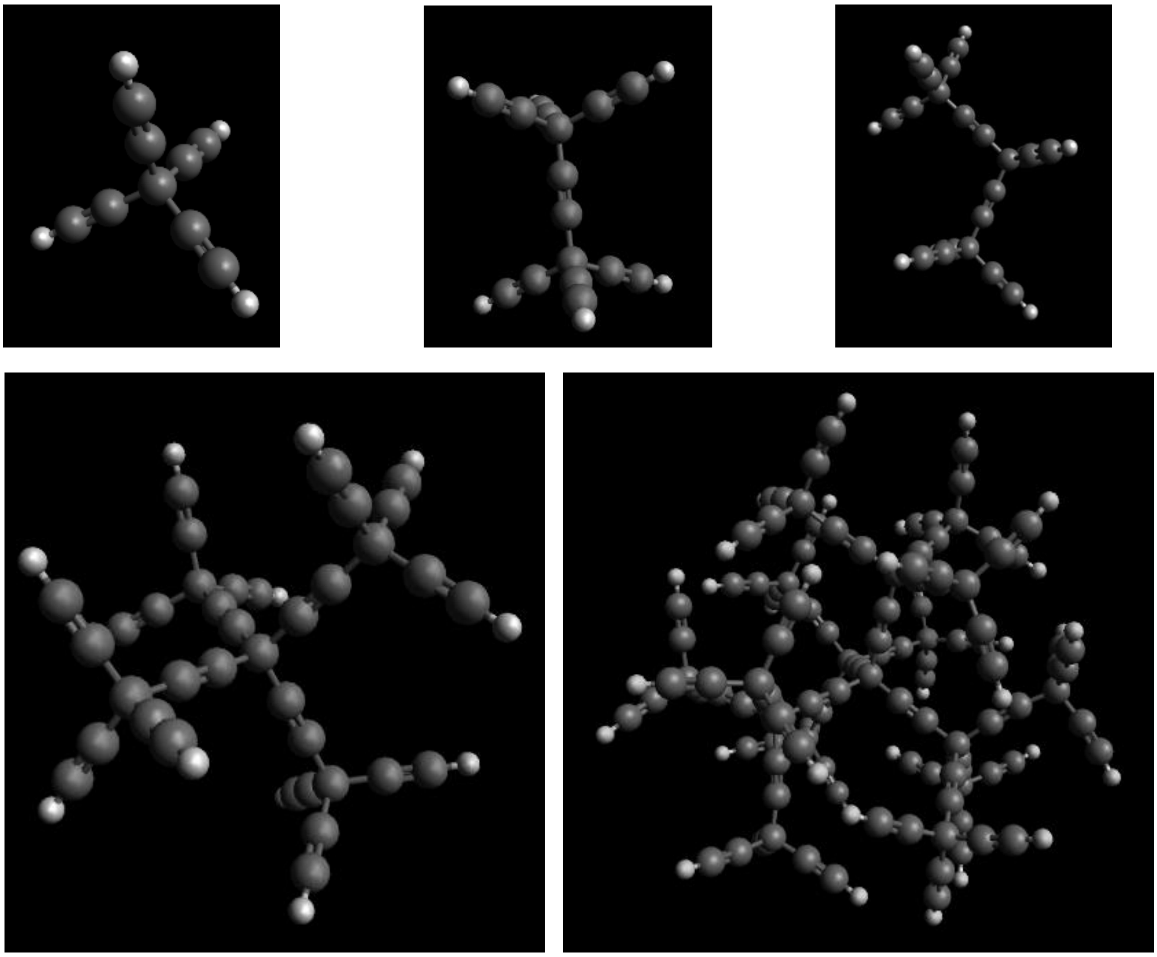

Figure S5: The OSPC-0 fragments used for gaussian calculations, Top R-L a monomer, 2 unit intermediate, and 3 unit intermediate. Bottom R-L a tetramer and octamer.

Table S2: The simulated Raman shifts in cm^{-1} of *sp*-*sp* stretching in inner and outer alkyne groups and of sp^3 stretching in the inner carbons. Due to the basis sets used and the lack of context for the wider environment around the fragments the simulated shifts are significantly different from the observed values, however the order in which the bands appear in both intensity and shift value was sufficiently consistent for our conclusions.

**Basis set**	***sp*-*sp* symmetric stretching – centre alkynes**				
**Structure**	Monomer	2u	3u	Tetramer	Octamer
**HF/STO**	2692	2861	2863	2865	2866
**HF/3-21G**	2435	2604	2607	2594	2620
**B3LYP/3-21G**	2270	2406	2409	2397	2423
**B3LYP/6-311+g(d)**	2406	2369	2371	2375	
**B3LYP/6-311+g(d,p)**	2406	2369	2371	2375	

**Basis sets**	***sp*-*sp* symmetric stretching – edge alkynes**				
**Structure**	Monomer	2u	3u	Tetramer	Octamer
**HF/STO**	n/a	2691	2690	2691	2690
**HF/3-21G**	n/a	2434	2607	2406	2431
**B3LYP/3-21G**	n/a	2267	2267	2241	2259
**B3LYP/6-311+g(d)**	n/a	2232	2232	2232	
**B3LYP/6-311+g(d,p)**	n/a	2232	2232	2232	

**Basis sets**	***sp*^{3}-*sp* stretching**				
**Structure**	Monomer	2u	3u	Tetramer	Octamer
**HF/STO**	1281	1278	1277	1197	1198
**HF/3-21G**	1166	1174	1176	1162	1109
**B3LYP/3-21G**	1062	1064	1021	995	996
**B3LYP/6-311+g(d)**	1175	1072	1021	994	
**B3LYP/6-311+g(d,p)**	1176	1073	1021	994	

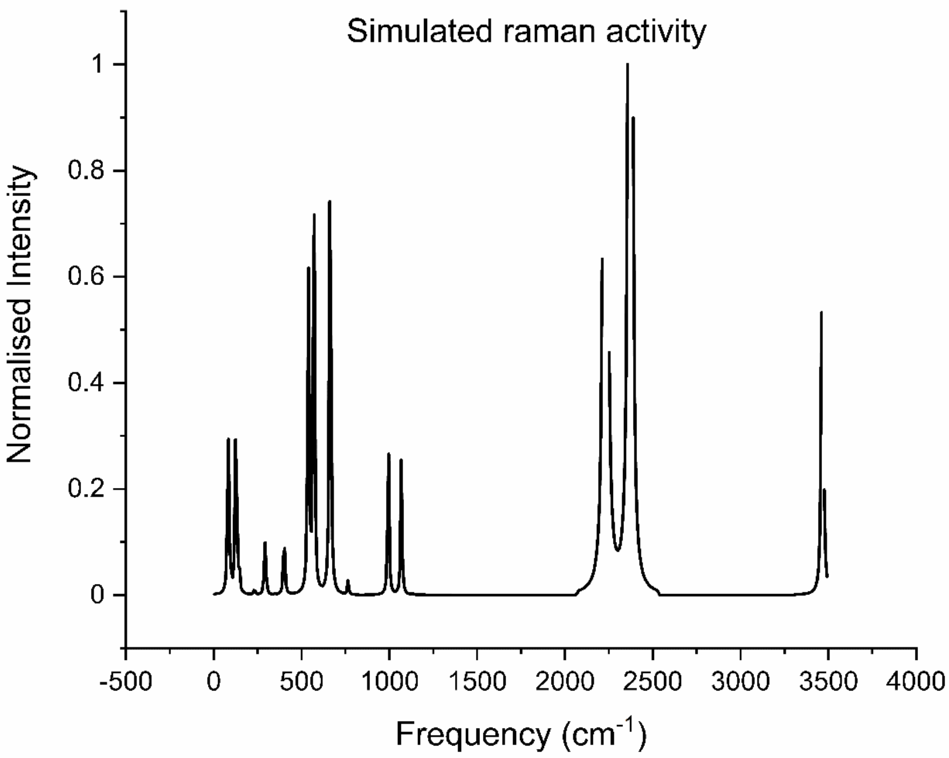

Figure S6: The full simulated Raman spectrum of the OSPC-0 tetramer calculated using the B3LYP/6-311+g (d, p) basis set, as it is a simulated spectrum it includes discontinuities. Many of the peaks, such as those at 3500 cm^{-1} and 100 cm^{-1} are from vibrational modes that would not occur in a rigid long-range structure so were excluded from our analysis. The three largest sets of peaks are from the sources described above.

8. Surface area analysis

Surface area analysis was undertaken for two samples of both OSPC-0 and OSPC-1. For the first sample, only uptake was acquired due to limitations of equipment. For the second sample, a more detailed full analysis was undertaken giving absorption and desorption isotherm and full pore volume analysis. The difference in values between the two samples respectively can be rationalised through the inherent errors of surface area analysis using the BET equation and the expected range due to the amorphous nature of the materials.

Table S3: Surface area data for OSPC-1 and OSPC-0 from the initial and repeat readings obtained at 77 K.

All units in m^2g^{-1}	OSPC-0 Initial	OSPC-0 Repeat	OSPC-1 Initial	OSPC-1 Repeat
BET Surface Area:	473	261	726	909
Langmuir Surface Area:	901	429	1380	1593
t-Plot Micropore Area:	244	65.7	430	617
t-Plot external surface area:	230	204.3	296	292
BJH Adsorption cumulative surface area of pores	164	170	198	200
BJH Desorption cumulative surface area of pores	N/A	155	N/A	151
D-H Adsorption cumulative surface area of pores	148	168	178	197
D-H Desorption cumulative surface area of pores	N/A	151	NA	145

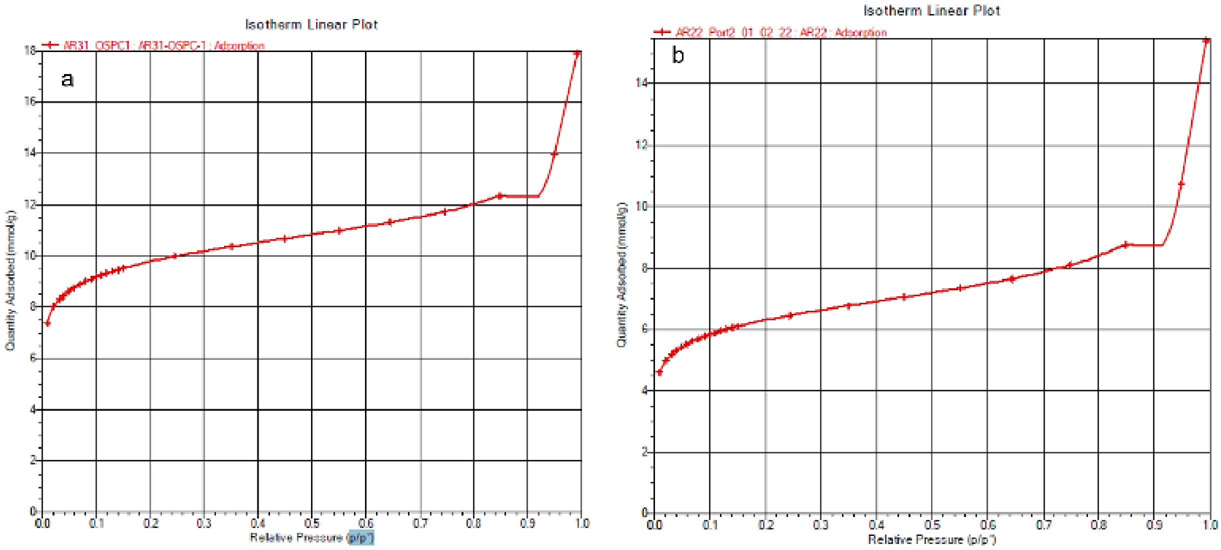

Figure S7: Initial N_2 adsorption isotherm of OSPC-1b (a) and OSPC-0b (b) at 77 K.

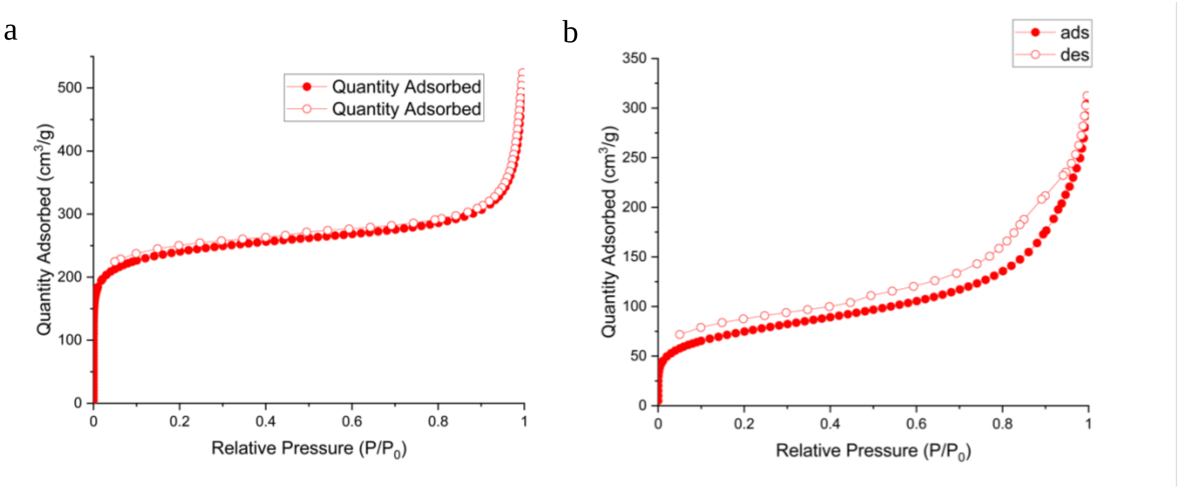

Figure S8: Repeat N_2 adsorption isotherm of OSPC-1b (a) and OSPC-0b (b) at 77 K.

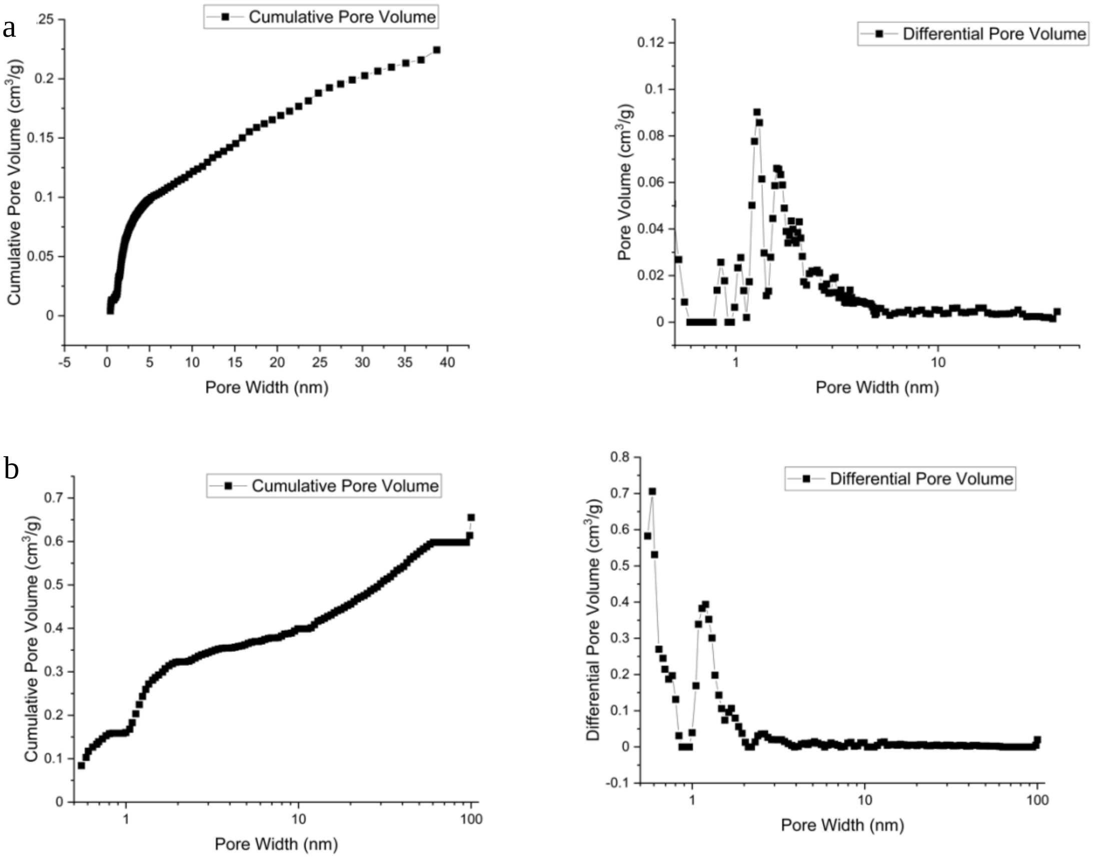

Figure S9: Cumulative and Differential pore volume plots of OSPC-0b (a) and OSPC-1b (b) at 77 K.

9. CHNS elemental analysis

Table S4: CHNS analysis of OSPC-1b and OSPC-0b. As the total is below 100%, the samples did not burn completely, so the percentage abundances are only representative of the parts of the sample that were burned. This also omits any Si or Br present, which are expected to be a significant minority component of the network.

Sample	C (%)	H (%)	N (%)	S (%)	Total (%)
OSPC-1b	43.72	2.832	0.1	1.248	47.9
OSPC-1b	43.71	2.458	0.31	0.746	47.224
OSPC-1b	59.03	3.423	0.95	1.648	65.051
OSPC-1b	59.9	3.401	1.12	1.578	65.999
OSPC-0b	47.55	2.525	0.79	3.672	54.537
OSPC-0b	49.19	2.424	0.9	3.974	56.488

10. X-ray Photoelectron Spectroscopy

Six spectra were obtained for OSPC-1b and five spectra were obtained for OSPC-0b. The C 1s spectra were fitted, with the sub peaks around 284.6 eV assigned to C-C *sp*^{3}, those around 285.2 assigned to C-C *sp*, and the peak around 286.7 assigned to C-Br in accordance with previously reported data.[4] The wide spectra were fitted to the appropriate elemental peaks with the reports.

Table S5: Carbon environment abundance data for OSPC-1b spectra in Figure 5.

Spectra	a	b	c	d	e	*x*	*σ*
C-C *sp*^{3} (%)	47.43	46.89	45.42	62.20	69.41	51.786	13.94
C-C *sp* (%)	49.94	50.68	51.18	33.11	26.74	55.566	14.32
C-Br *sp* (%)	2.62	2.44	3.4	3.85	3.85	3.648	0.75

Table S6: Carbon environment abundance data for OSPC-0b spectra in Figure 6.

Spectra	a	b	c	d	e	f	*x*	*σ*
C-C *sp*^{3} (%)	65.05	46.89	60.08	55.56	49.78	54.20	55.26	6.63
C-C *sp* (%)	33.42	50.68	38.52	42.39	47.77	44.91	42.95	6.28
C-Br *sp* (%)	1.53	2.44	1.39	2.05	2.45	0.89	1.79	0.62

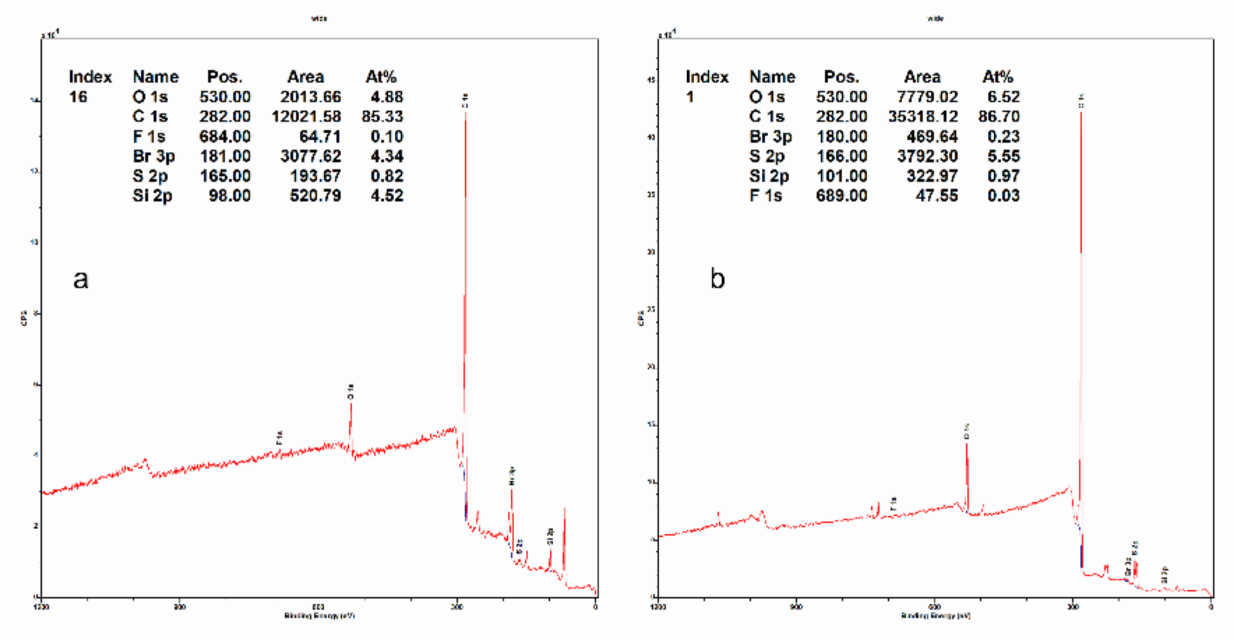

Figure S10: Example wide XPS spectra of OSPC-1b (a) and OSPC-0b (b).

Table S7: Wide spectrum XPS data for OSPC-1b.

Element\Spectra	a	b	c	d	e	*x*	*σ*
O (%)	3.50	4.66	3.47	3.66	4.88	4.03	0.68
C (%)	86.19	84.94	85.80	85.73	85.33	85.60	0.48
F (%)	0.57	0.25	0.33	0.52	0.10	0.35	0.19
Br (%)	4.59	4.81	4.79	4.86	4.34	4.68	0.26
S (%)	0.76	0.77	0.81	0.94	0.82	0.82	0.07
Si (%)	4.38	4.56	4.8	4.29	4.52	4.51	0.20

Table S8: Wide spectrum XPS data for OSPC-0b.

Element\ Spectra	a	b	c	d	e	f	*x*	*σ*
O (%)	6.52	6.11	5.89	5.45	6.72	6.31	6.16	0.42
C (%)	86.73	87.32	87.84	88.19	87.33	87.23	87.44	0.51
F (%)	0	0	0.15	0.11	0	0	0.04	0.06
Br (%)	0.23	0.11	0.03	0.07	0.19	0.11	0.12	0.07
S (%)	5.55	6.13	5.82	6.17	5.76	6.21	5.94	0.27
Si (%)	0.97	0.34	0.26	0	0.01	0.14	0.29	0.36

11. SEM and EDX

The EDX results for OSPC-1b showed a greatly elevated level of Br compared to the XPS data (table S17). We do not see direct evidence of high bromine levels in the wide XPS spectra, nor indirect evidence of C-Br in the carbon XPS and carbon NMR. It is therefore likely that the readings are anomalous, either due to the sample submitted, or to the nature of OSPC-1 or OSPC-1b itself as a *sp* based carbon material. The OSPC-0b spectra are more in line with expectation; however, they show elevated levels of sulphur, likely from the DPS solvent. All samples were washed in the same way, so possibly the more tightly packed OSPC-0b network makes it more difficult for the solvent molecules to wash out after network formation.

Table S9: EDX data for OSPC-0b and OSPC-1b.

		**Abundance (%)**						
	Element	Site 1	Site 2	Site 3	Av	SD		
**OSPC-0b**	C	83	83.2	82.8	83	0.20		
	S	7.7	6.1	6.1	6.6	0.92		
	O	7	8.1	9	8.03	1.0		
	Cs	1.2	1	0.9	1.03	0.15		
	Na	0.4	0.4	0.4	0.4	0		
	Br	0.4	0.3	0.2	0.3	0.1		
	Si	0.3	0.4	0.5	0.4	0.1		
		Abundance (%)						
	Element	Site 1	Site 2	Site 3	Site 4	Site 5	Av	SD
**OSPC-1b**	C	65.8	65.1	65	71.5	65.7	66.6	2.75
	Br	24.2	25.3	25.2	19.4	24.4	23.7	2.45
	Si	4.7	4.5	4.9	4.9	5.3	4.7	0.30
	O	3	2.9	2.9	2.6	2.8	2.8	0.15
	S	1.4	1.3	1.3	1.1	1.2	1.3	0.11
	F	0.7	0.6	0.6	0.4	0	0.5	0.28
	Cl	0.4	0.3	0.2	0.1	0	0.2	0.16

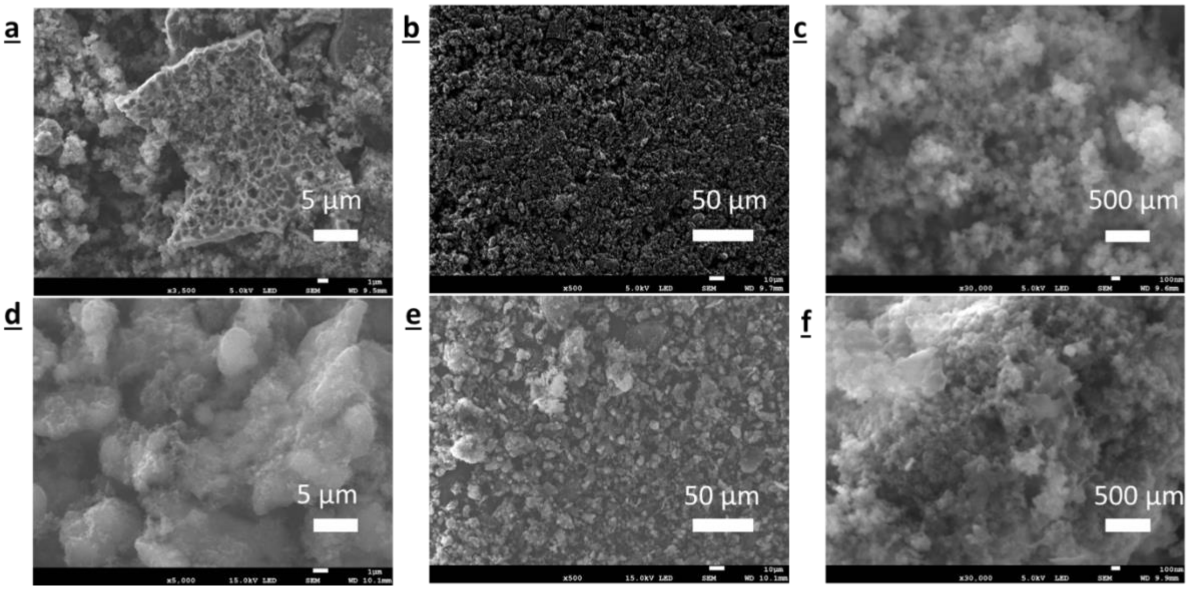

Figure S11: SEM images of OSPC-1b (a, b, c) and OSPC-0b (d, e, f).

12. Electrochemical analysis

As our electrodes and coin cells were prepared by hand, there was significant variability in the mass of active material present on an electrode and the morphology of the electrode surface. Additionally, as OSPCs are new materials there is no established practice for formulating slurries and controlling their rheology. This resulted in significant variability in the specific capacities observed during galvanostatic cycling, shown in Figure S12. However, the shape of the charge discharge curve was the same for all cells. Reproducibility could be improved by mechanising the electrode casting and cell assembly process to ensure every cell is optimal, and by investigating how OSPCs behave in slurries and gels, however this would require specialist equipment that is beyond the scope of a pilot investigation. Our headline capacity figures are from the cells that gave the highest specific capacity at 100 cycles, as these are most representative of a hypothetical commercial cell made under optimised conditions. At a fixed current density, the cells with greater specific capacities took longer to cycle, so data out to 1000 cycles was only available for cells with lower specific capacities. However, as no degradation from the lower reversible cycling capacity was observed during the cycling of the cell we believe it is representative of the general trend in performance of OSPC-1 cells during long term cycling. Cells for all other tests were used and reported as they were made without any selection for capacity. The lower voltage window of 0-2 V rather than 0-3 V was chosen after some preliminary cells exhibited degradation above 2 V that appeared to be from oxidation of the electrolyte. This behaviour was not observed in subsequent cells, but we maintained the window to allow comparison with earlier work.

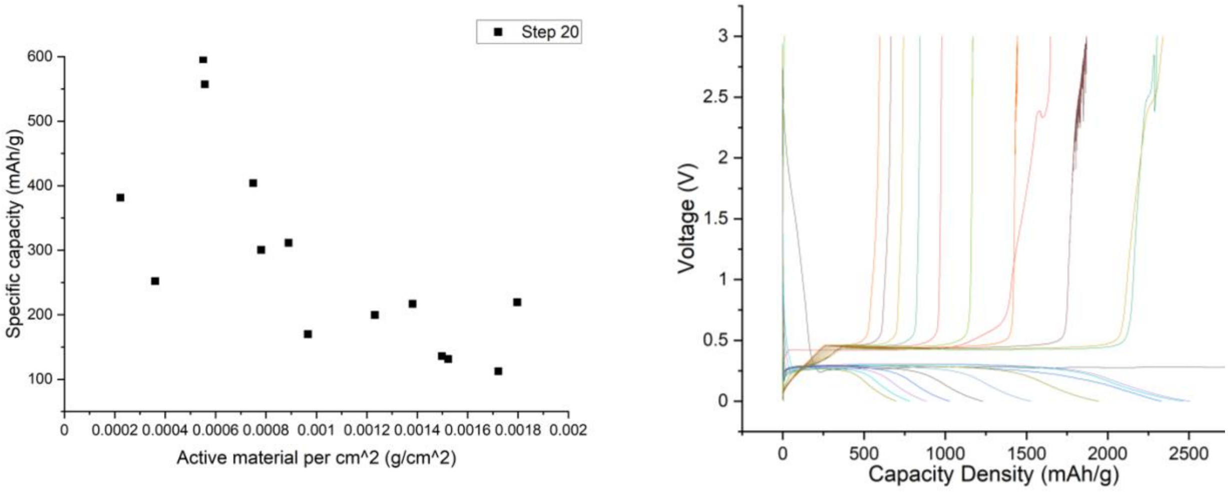

Figure S12: A scatter plot showing the variability observed in OSPC-1b cells, and the galvanostatic cycling curve of the very first OSPC cell made. We are unsure why the first few cells were so unlike every subsequent cell.

To investigate if cycling at a higher voltage window would yield greater capacities, we cycled an OSPC-1b and an OSPC-0b cell between 0-3 V under the same conditions as the cells cycled between 0-2 V. We did not observe an increase in capacity, nor any significant difference in the charge/discharge curve. We therefore believe that the results we obtained between 0-2 V are comparable to the literature results obtained between 0-3 V.[1]

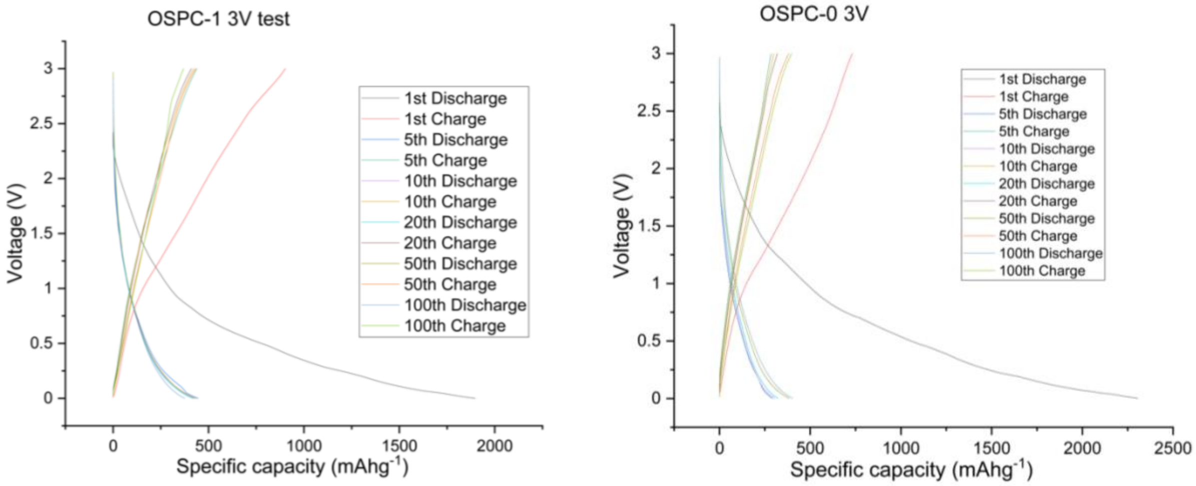

Figure S13: The galvanostatic cycling data for OSPC-1b and 0b cells cycled between 0 and 3 V.

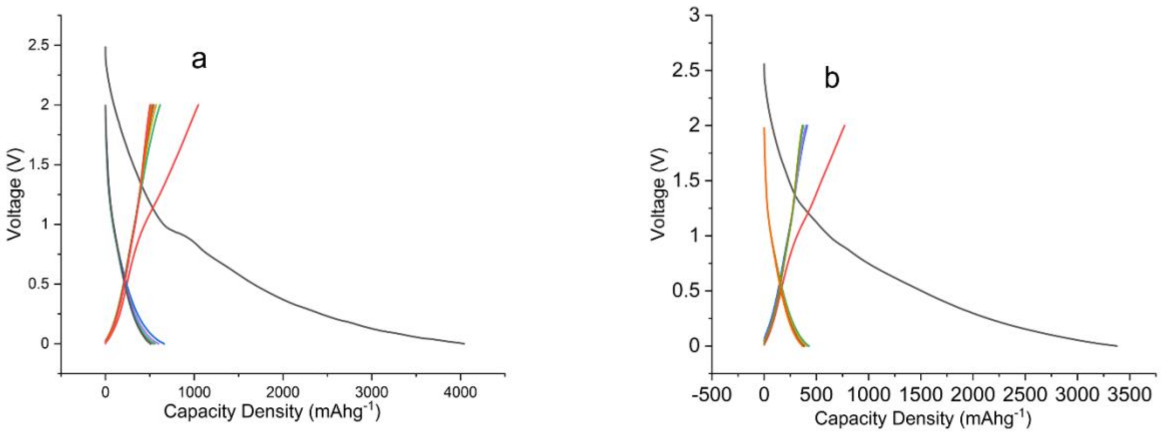

Figure S14 The galvanostatic cycling data for OSPC-1b and 0b cells cycled between 0 and 2 V.

13. SEM Over-discharge investigation

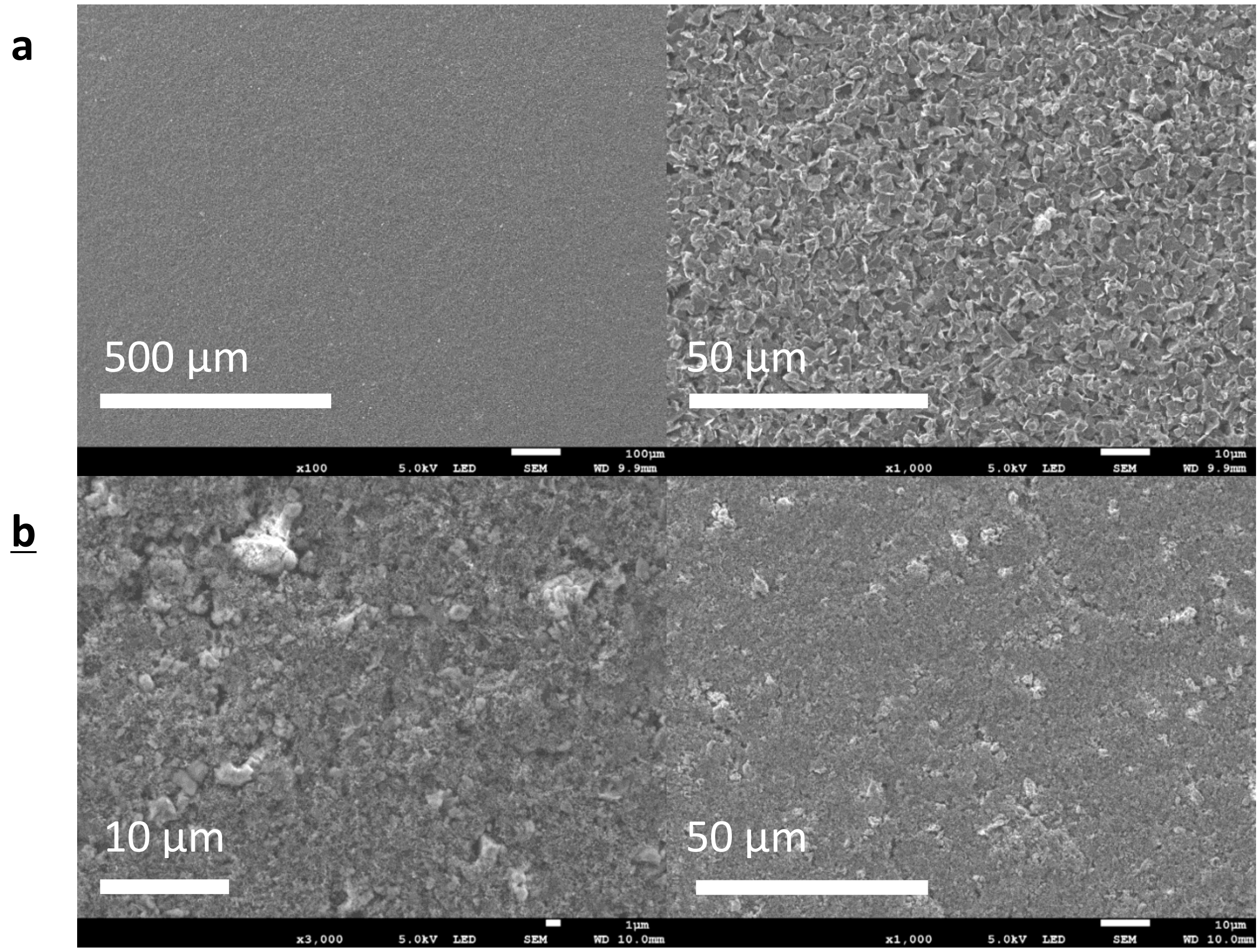

Figure S15: SEM images of a pristine graphite electrode (a) and a pristine OSPC-1b electrode (b).

14. XRD stress test investigation

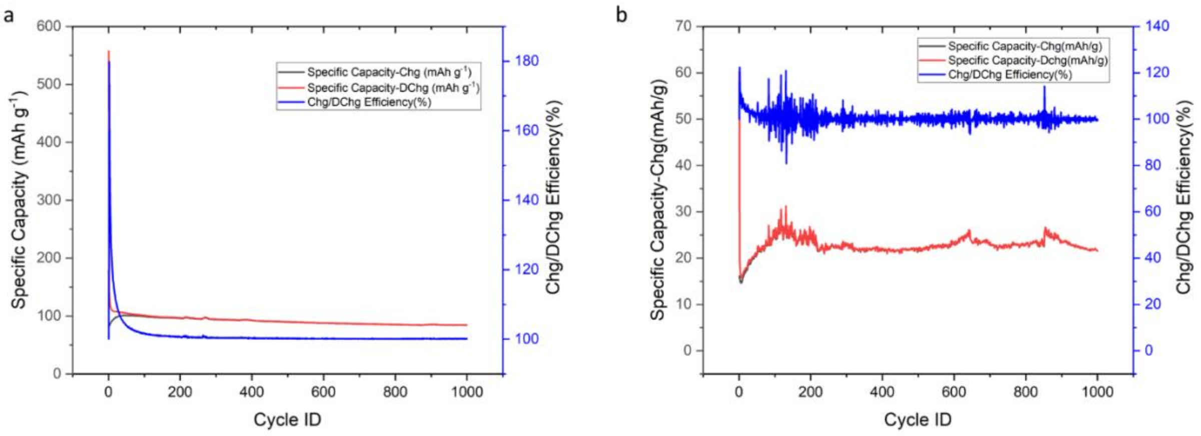

Figure S16: Cycling data for OSPC-1b (a) and graphite (b) electrodes from the stress test cycling pattern of 1000 cycles at 5000 mA g^{-1}.

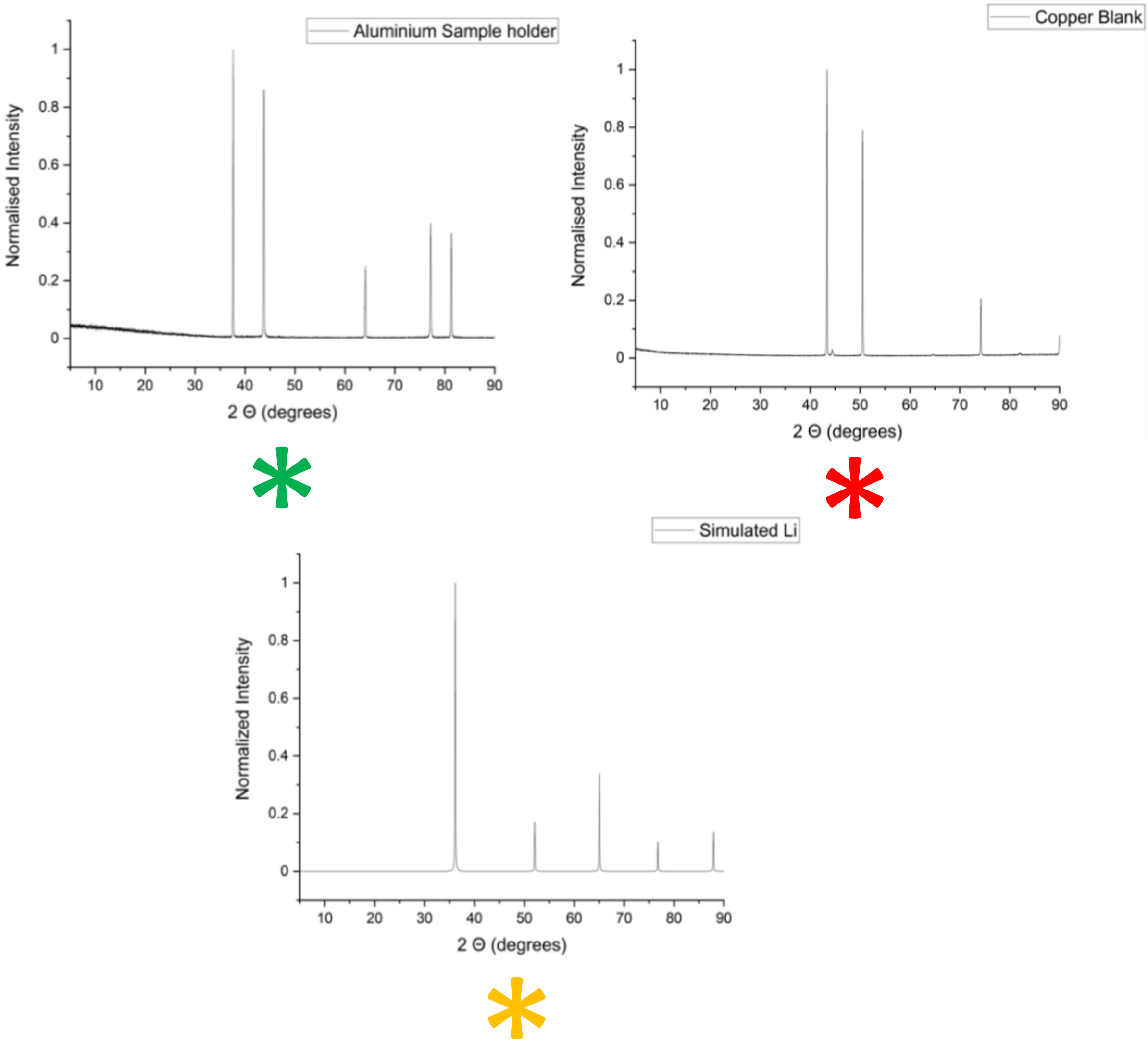

Figure S17: XRD patterns of the aluminium sample holder (green) and copper current collector (red) with a simulated XRD pattern of metallic lithium (yellow). These were used to assign the subsequent patterns, with the coloured asterisks referring to these materials.

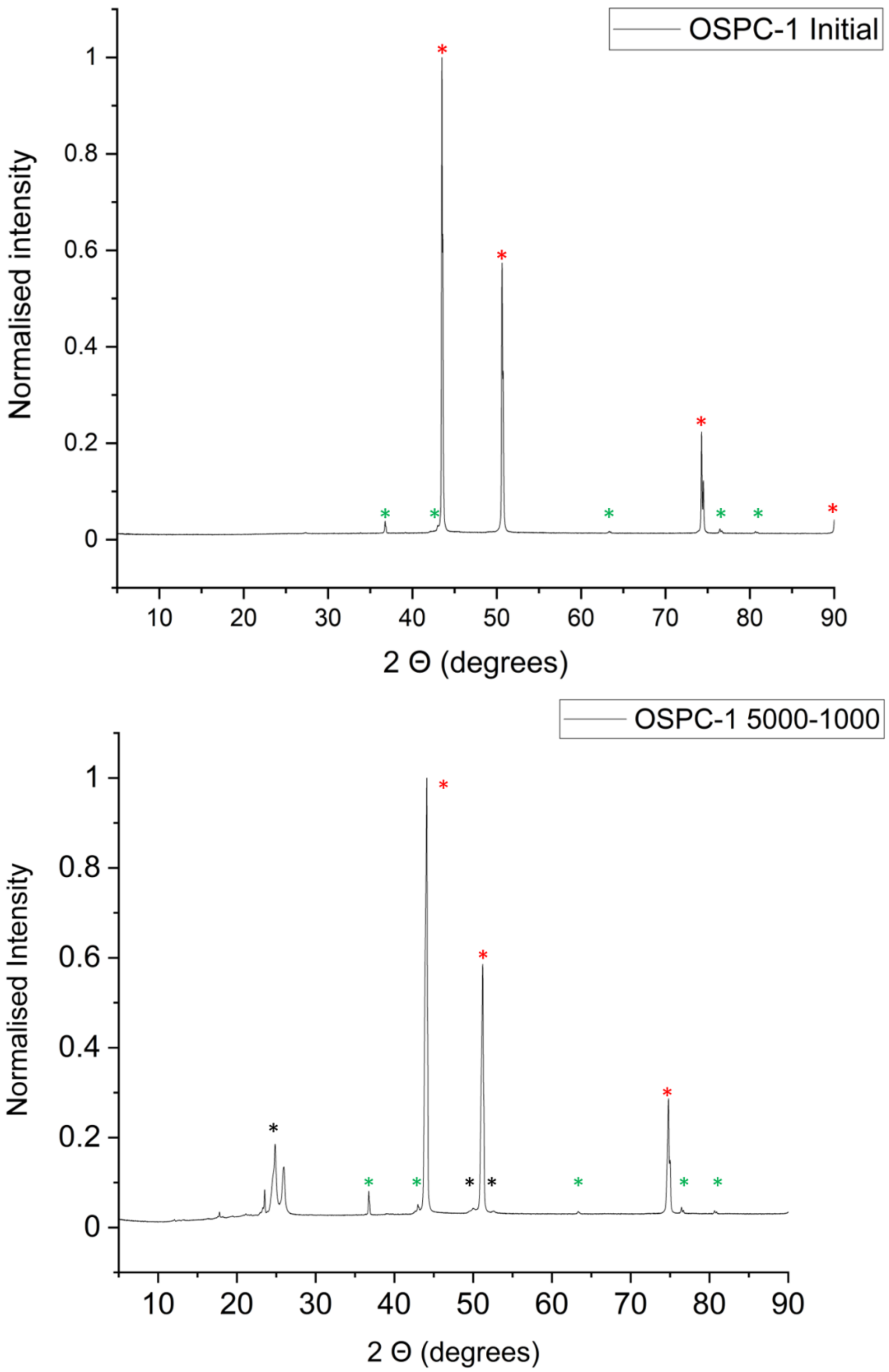

Figure S18: XRD patterns of a pristine OSPC-1b electrode (top) and a stress-tested electrode (bottom). As OSPC-1b is amorphous it cannot be observed by XRD, but degradation of OSPC-1b and or the carbon black into graphite and graphite like structures is observed (black asterisks).[5]

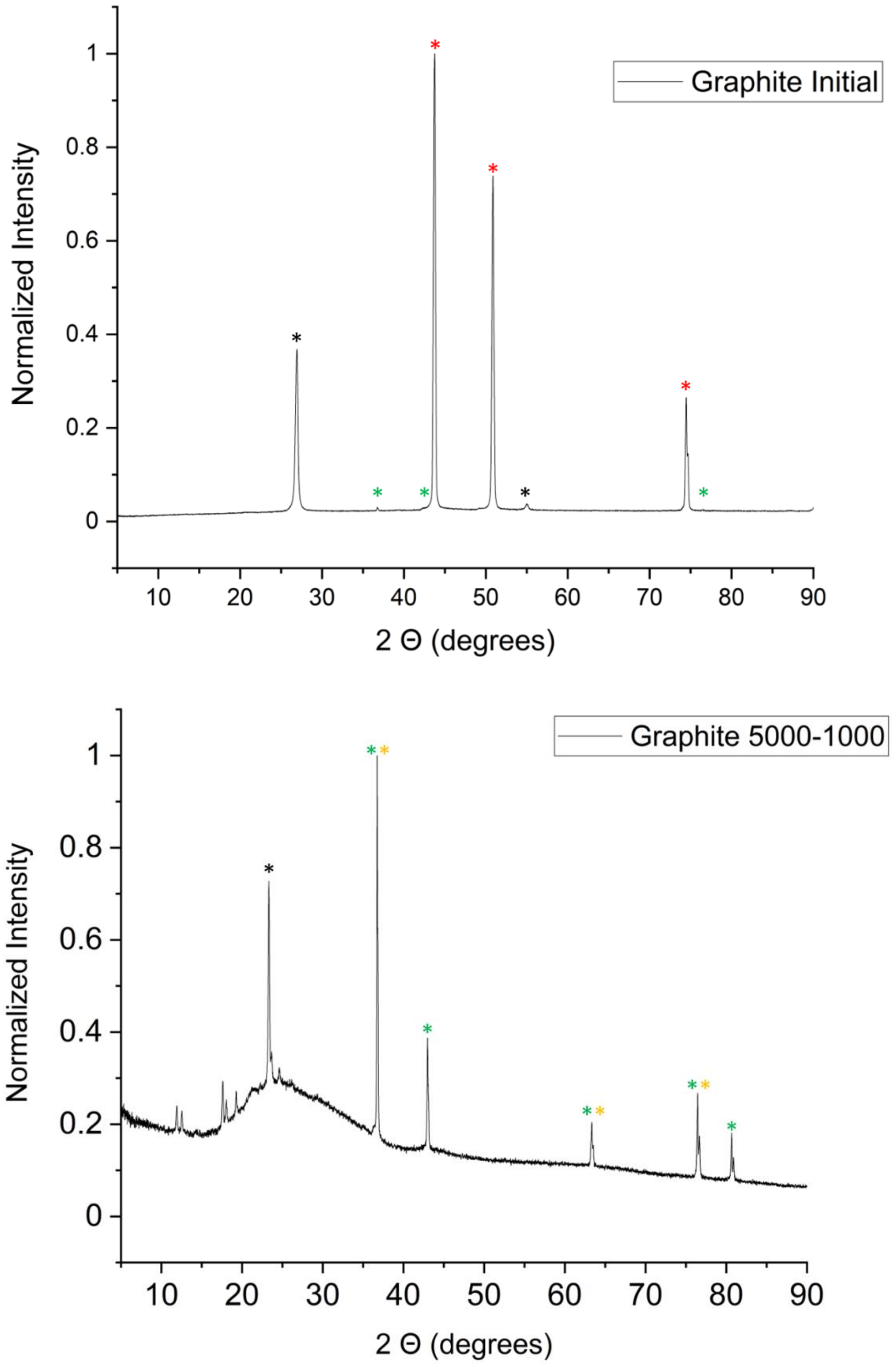

Figure S19: XRD patterns of a pristine graphite electrode (top) and a stress tested electrode (bottom). In the pristine electrode graphite peaks are visible at 27˚ and 65˚ 2θ. In the stress tested electrode, the copper current collector detached from the electrode during cell

disassembly so is not observed. The aluminium sample holder has similarly positioned peaks to the calculated metallic Li spectra. Whilst we can see shoulders on the shared peaks, we cannot determine which material is the peak and which is the shoulder. We attribute the distortion of the graphite peak at 27° 2θ to disruption of the graphite lattice structure from the intercalated lithium and the effect of repeated intercalation and deintercalation of lithium from the stress test's conditions.[6] The smaller peaks observed between 10° and 30° 2θ could be from LiC_x , such as LiC_{60}, formed during the degradation of the lattice.[7] If the carbon black is the source of the graphitisation in the OSPC-1b electrode we would also expect to see it here, but if it is present it is obscured by the degraded graphite structure.

15. References

(1) Zhao, Z.; Das, S.; Xing, G.; Fayon, P.; Heasman, P.; Jay, M.; Bailey, S.; Lambert, C.; Yamada, H.; Wakihara, T.; et al. A 3D Organically Synthesized Porous Carbon Material for Lithium-Ion Batteries. *Angewandte Chemie International Edition* **2018**, *57* (37), 11952-11956. DOI: 10.1002/anie.201805924.

(2) Mollart, C.; Trewin, A. Conjugated microporous polymer frameworks for sustainable energy materials – elucidating the influence of solvents on the porosity properties for future design principles. *Journal of Materials Chemistry A* **2024**, 10.1039/D3TA04866G. DOI: 10.1039/D3TA04866G.

(3) Mollart, C.; Trewin, A. Rationalising the influence of solvent choice on the porosity of conjugated microporous polymers. *Physical Chemistry Chemical Physics* **2020**, *22* (38), 21642-21645.

(4) Wang, J.; Fu, X.; Yan, N.; Zhang, Y. Molecular Design of 3D Porous Carbon Framework via One-Step Organic Synthesis. *ChemSusChem* **2021**, *14* (18), 3806-3809. DOI: https://doi.org/10.1002/cssc.202101262.

(5) Li, Z. Q.; Lu, C. J.; Xia, Z. P.; Zhou, Y.; Luo, Z. X-ray diffraction patterns of graphite and turbostratic carbon. *Carbon* **2007**, *45* (8), 1686-1695. DOI: https://doi.org/10.1016/j.carbon.2007.03.038.

(6) Yuan, F.; Hu, J.; Lei, Y.; Zhao, R.; Gao, C.; Wang, H.; Li, B.; Kang, F.; Zhai, D. Key Factor Determining the Cyclic Stability of the Graphite Anode in Potassium-Ion Batteries. *ACS Nano* **2022**, *16* (8), 12511-12519. DOI: 10.1021/acsnano.2c03955.

(7) Gaboardi, M.; Duyker, S.; Milanese, C.; Magnani, G.; Peterson, V. K.; Pontiroli, D.; Sharma, N.; Riccò, M. In Situ Neutron Powder Diffraction of Li6C60 for Hydrogen Storage. *The Journal of Physical Chemistry C* **2015**, *119* (34), 19715-19721. DOI: 10.1021/acs.jpcc.5b06711.
